# Accounting for peoples’ preferences in establishing new cities: A spatial model of population migration in Kuwait

**DOI:** 10.1371/journal.pone.0209065

**Published:** 2018-12-13

**Authors:** Nayef Alghais, David Pullar, Elin Charles-Edwards

**Affiliations:** School of Earth and Environmental Sciences, The University of Queensland, St Lucia, Brisbane, Queensland, Australia; Pavol Jozef Safarik University in Kosice, SLOVAKIA

## Abstract

Modelling of internal migration to new cities is challenging, yet necessary to ensure that these newly established urban areas will be populated and function as intended. In the State of Kuwait, there is a unique set of push and pull factors: government subsidised housing for citizens, the existence of a single urban area, and the initiation of a new and ambitious master plan for the construction of 12 new cities, which are expected to attract not only locals, but also international residents and businesses. On top of these factors, there is an unusual demographic situation, as non-citizens outnumber Kuwaiti citizens by a factor of 2.3, with these groups having widely different preferences in terms of housing. Currently, there is no plan to take these resident groups’ opinions into consideration for the new cities project. Besides, the current study simulates the impacts of the involvement of residents in urban planning. Samples from resident groups (citizens and non-citizens) participated in targeted surveys and useful answers were extracted in relation to the migration likelihood, push and pull factors that may affect their decisions, spatial preferences for new cities and their opinions on segregation by nationality. Specifically, the survey results showed significant interest of residents in moving to the new cities. For citizens, the most important factors in deciding whether to move or not were proximity to their close family and housing availability, while for non-citizens the most important factor was the creation of new employment opportunities. Both survey groups agreed that existing city property prices are too high and make the prospect of moving to a new city more attractive. The responses were transferred in an Agent Based Model, and the simulations showed certain differences to the official projections for 2050 without the public responses, in regards to the geographical distribution of the most desirable suburbs. Furthermore, the simulations showed that in the new cities, nationality segregation levels are expected to drop by at least 15% compared to the 2015 levels. The findings may be utilised by the authorities to modify the master plan accordingly.

## 1. Introduction

Kuwait is an Arabian Gulf country that has experienced a rapid population growth in the last decades mainly driven by international migration [1]. This has led to the suggestion that new cities be established in the region, which in turn raises questions about the optimal location of the cities, their desirability by the locals, and whether they would result in migration from all segments of the population.

Predicting migration and future population distribution are essential for the successful development of new urban areas, the provision of affordable housing and creation of new job opportunities [2]. The majority of migration research conducted in industrialised and developing countries focus on the economic concerns for metropolitan areas experiencing urban growth; such as New York [3], Auckland [4] and Delhi [5]. The underlying driver of the movement of people is mostly economic, especially for new employment opportunities [5–7].

In Arabian Gulf countries and specifically in Kuwait, economic-driven migration may not be significant due to its plentiful resources (oil and natural gas) and relatively small size leading to relatively uniform distribution of wealth. In fact, the drivers behind internal migration in Kuwait and similar Arabian Gulf countries as a geographical phenomenon are largely unknown. The reason for this is mainly the lack of data about internal migration. In the past, urban growth in Gulf cities was mainly driven by city expansion and intensification and no independent cities were developed [8, 9], which suggests that the drivers of internal migration could differ substantially from other industrialised societies. In these situations, internal migration was limited in magnitude and when present, it was over very short distances and between regions with minor differences; hence the lack of migration data was not causing any profound negative impacts.

Recently however, and as many new cities are planned to be constructed in Arabian Gulf countries [10, 11], the lack of internal migration data is becoming a more serious issue. The motivations and decisions of residents to move to new urban areas must be understood in order to guarantee the project’s success. Towards that goal, collecting data directly from a survey targeting residents in Kuwait is a novel solution implemented in this paper. The survey responses can provide important new insights into internal migration in Arabian Gulf countries. Additionally, the survey results can also be used to predict and simulate the internal migration in the case study city of Kuwait and provide a more practical view on the new cities future state. Finally, and in order to validate the findings with a theoretical background, the responses will also make it possible to identify the main reasons behind resident choices in the context of push and pull factors theory.

Push-Pull theory of migration is used to identify the factors driving migration at origins and destinations [12]. Traditional urban theories on the drivers of internal migration, which are linked to economic trends may not be relevant in Kuwait’s case and such as alternative urban theories are employed instead to explain resident settlement preferences [13, 14].

In modern industrialised societies, it is common for government planning authorities to regulate push and pull factors, for instance by offering new public sector jobs or subsidising housing markets in new planned cities in order to achieve a balanced population distribution [15, 16]. In the modern era internal migration is usually more subtle by providing indirect incentives to potential movers [17].

Urban planning that does not thoroughly consider the drivers of internal migration may result in costly and unsuccessful urbanisation policies. Generally, this is not an issue in cases where development planning is transparent and conducted in harmony with the market trends and community participation [18, 19]. However, in countries with centralised planning there may be little in terms of public engagement so making predictions about internal migration is speculative at best. In the case of Kuwait, the involvement of the public in planning and land use decisions is limited or in most cases non-existent. The implications of the lack of public involvement in Kuwait (or any similar case) may lead to:

- Lack of sufficient number of people migrating to new cities and hence resulting in uninhabited cities, or
- Stagnated growth for new cities if a lower than expected numbers of people settle in the new cities.

One notable example of the risks of excluding the public opinion in planning is the construction of Ghost Cities in China; complete cities established with full infrastructure, but without any residents [20]. It can be argued that if the Chinese government had involved residents in the planning process and before any decisions were made, Ghost Cities may not have occurred, as there would be a solid understanding of the internal migration trends and preferences of residents. Adding to the problem, largely heterogeneous population groups with different preferences and needs may respond very differently in regards to push or pull factors. Settling new cities raises other issues such as achieving socially integrated communities; this is typically described as segregation by wealth, race or nationality. In Kuwait, segregation by nationality is prevalent due to land use policies.

The Kuwait government is launching a project for developing new cities for its current and future residents [21]. This study recognises and investigates this research opportunity related to assessing the importance of public participation in urban development, identifying the push and pull factors and modelling the impacts of establishing new cities on residential segregation. This paper attempts to study future internal migration in Kuwait, which we believe typifies trends in other Arabian Gulf countries. The research questions addressed in this paper are:

- Will future planned city areas attract sufficient population for settlement?
- Do people’s preferences on migration and future cities differ from the government plans, and potentially avoid problems of creating ghost cities?
- Will removing land use policies that separate Kuwaitis and non-Kuwaitis change future settlement patterns?

This will be done by simulating the migration to new cities in Kuwait under different scenarios, including scenarios where public opinion for urban development is considered. The preferences and responses of the two resident groups (citizens and non-citizens) in Kuwait in relation to housing and migration obtained via an online survey and are integrated as behaviours in a simulation model. Finally, simulation outcomes are assessed in terms of their effectiveness in addressing a key urban issue in Kuwait, namely the level of residential segregation.

## 2. Background

### 2.1 The value of participatory planning in urban development

Among the most important issues in urban planning is failing to meet project goals due to differences between community stakeholders’ perspectives, especially when resident opinions are not included in planning [22]. In order to prevent this, many cities have amended their systems to involve the public in the planning and decision making processes of urban development. This may be done through consultations with community committees, open forums, public meetings and citizens’ surveys [23].

The aforementioned methods are part of a planning school referred to as participatory or comprehensive planning. Comprehensive planning, as the name implies, involves a holistic view on urban planning that accounts for the needs of broad demographic groups as a whole, rather than just planning for individual and/or localised urban systems, like residential zones or motor networks. Decisions for the medium and long term in comprehensive plans are made with consideration of multiple factors at the same time, ranging from land use to infrastructure and transportation networks and how these factors interact with each other. In comprehensive planning, community participation in decision making is vital to ensure that the needs of all resident groups and urban stakeholders are considered. Participatory planning involves the residents in the decision making processes in an attempt to develop successful urban development [24]. The benefits of participatory planning include promoting the notion of democracy, and validating the decision making processes. However, there are also disadvantages, such as increased complexity and costs. Projects with participatory planning are mainly limited to small scale development [25, 26]. Integrating public opinion at neighbourhood level may be done through public surveys or web questionnaires [27]. For large scale regional development projects in cities, state or national level, the decision making is centralized and state controlled, with inputs from studies and consultations with experts [23, 28]. Kuwait does not have a framework of comprehensive planning, but as a country it is small enough to conduct a form of participatory planning with public engagement conducted via a tailored online survey, the details of which are presented in section 3.

### 2.2 Modelling internal migration to new cities

Several internal migration models have been proposed in recent decades based on the individual migrants’ behaviours and decision making. Some models attempt to simulate migration based upon theorised human-economic behaviour, while others are more empirical and rely on direct observation or collected data from key stakeholders and demographics [29]. In this paper a empirical internal migration model is developed based on the outcomes of a resident survey, which in turn attempts to identify push and pull factors driving migration.

Modelling internal migration to evaluate and check the validity of future development decisions can be done through simulating future locational patterns of migrant destinations [30]. The majority of urban models deal with small scale development projects, such as selection of residential housing [31, 32]. These urban residential-choice models may use questionnaires to generate outcomes of future residential movements based on the respondent’s preferences [33]. Another approach towards migration modelling (and particularly for transportation modelling) includes Discrete Choice Models (DCE). In DCE agents make decisions within the model space and among various constrained options, with an ultimate aim to maximise utility in regards to a range of variables. Migration modelling may also be carried out with the help of Agent Based Modelling (ABM) [29]. ABM is considered as one of the most suitable options, as it can convert small scale behaviours to larger scale spatial outcomes [34, 35].

ABM has been widely used for modelling urban planning and future development assessment around the world [36–41]. The effectiveness of ABM is due to its ability to capture local interactions within urban systems from a bottom-up perspective and its flexibility, particularly in terms of geospatial model development [35]. Furthermore, interactions between autonomous agents (individuals or groups) in ABM are examined at a disaggregate level, which reveals emergent macroscopic land use patterns. Another notable advantage is the capacity to model relations between agent decisions and actions, effects of heterogeneous values, and to downscale feedback from macro to micro-scale levels with the ability of adaptation in decision making [41]. The use of ABMs in planning and urban systems research can be beneficial as it both enhances the understanding of urban dynamics and helps predict a future system [42]. Furthermore, ABM can easily incorporate local interactions and rules for the agents (such as residents, developers or urban planners) involved in urban development. This is significant, as often goals of a certain group are constraints of another. Thus, ABM’s ability to generate future predictions and analyse trends with a bottom up approach can help in analysing the complexity of city systems and the dynamics therein.

Scheduling agent behaviours and decisions may take place over a period of time that could range from seconds to decades. The environment within agents live, commute and interact can also be simulated at various spatial and temporal scales [43]. The advantages of ABM include these abilities: to simulate stakeholder (agent) decisions and preferences as actions and interactions between the agents to assess ‘what if’ scenarios, to simulate micro level interactions within dynamic environments from a bottom-up perspective, to handle heterogeneous agents, and its flexibility in terms of geospatial model development [41, 44]. Due to the successful implementation of ABM in numerous studies, it was selected to predict the future impact of the master plan in Kuwait. Among the key reasons that ABM was deemed most suitable for use within this paper is that the authors aim to understand the nature of resident preferences in regards to migration, rather than relying on certain socio-economic heuristics to reflect their decision. In other words, the ABM reveals more about the cohort of population who migrate and their preference structure compared to other models like DCE.

Spatial modelling with ABM’s is important in urban planning for understanding the way cities grow with internal urban intensification or outward expansion, and for assessing potential negative impacts such as traffic congestion and housing shortage [9, 45]_4i7ojhp; This paper combines the use of ABM’s with GIS to model the establishment of new cities. The rules for the agent groups in the ABM are derived from questionnaire responses. There are three scenarios simulated in the ABM:

1. Government scenario: Simulates the urban development of new cities based on the government’s plans alone (without any public participation). Segregation is simulated according to the business as usual approach.
2. Resident scenario: Simulates urban development of new cities based on the resident preferences as extracted from the online survey. Segregation is simulated according to the resident responses.
3. Global Cities scenario: Simulates urban development of new cities based on the resident preferences as extracted from the online survey. Segregation is simulated according to the Global Cities plan (no segregation in new cities, only mixed districts).

For each of the above scenarios, nationality segregation levels and the potential of limited internal migration to new cities will be assessed. The outputs are expected to help in evaluating the new master plan and understanding the future internal migration trends in Kuwait.

### 2.3 Case study background

Kuwait is used as case study in this paper. Kuwait has a population of 4.5 million living in an area of 17,818 km2 and the non-citizens make up for 70% of the total population [46]. It should be noted that obtaining Kuwaiti citizenship is an extremely difficult process, involving a range of specific social, religious, work and family status conditions to be met. In general, only Arabic speaking, Muslim non-citizens that have close family ties or significant work experience in Kuwait are eligible to become citizens after a long period of time. This limited social mobility has significant impacts on land use and spatial distribution of the population.

**Fig 1:**
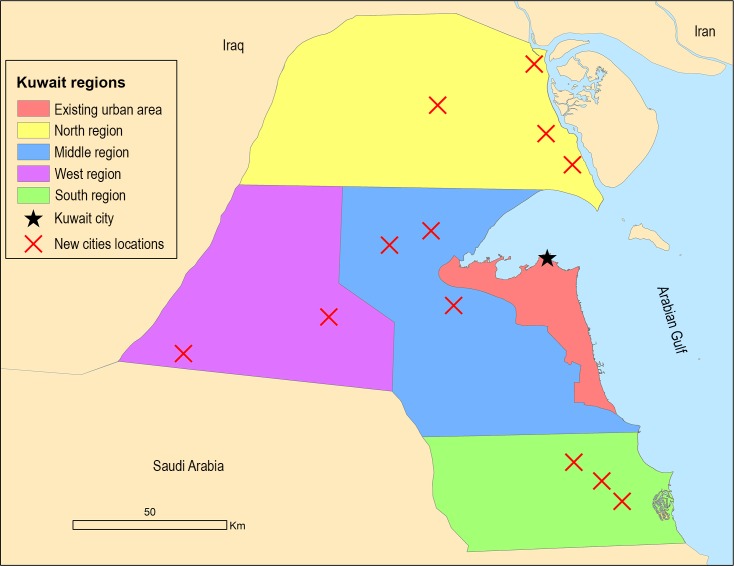
Kuwait regions and proposed cities locations.

The government plans to establish 12 new urban centres independent from the sole existing urban area of Kuwait City, but as of the date of this paper none as yet have been implemented [47]. In 2007 the government established a program titled “Kuwait Vision 2035” in order to transform Kuwait as the major financial and trade centre of the region by 2035 [48]. A major component of this plan is the development of new transportation modes, including a train network to link the old urban area with the new cities [49]. Fig 1 shows the new cities locations.

The most important issues identified for developing the new plan according to Kuwait government were housing shortages and traffic congestion [50]. According to the planning authorities, future development decisions were made without any input from the residents. Another distinct issue in Kuwait is the high residential segregation between citizens and non-citizens. This nationality segregation is due to the following reasons [51, 52]:

- The government policy of offering free dwellings to citizens in specific residential districts.
- The right to own a house is limited to Kuwaitis and Arabian Gulf citizens only Non-citizens may only rent or share a house and cannot legally own any property in the country [53].
- The differences in district type and dwelling type preference between citizens and non-citizens: Kuwaitis prefer residential districts and large plot size houses; whereas, non-Kuwaitis prefer mixed use districts and apartments [9, 53, 54].

**Fig 2:**
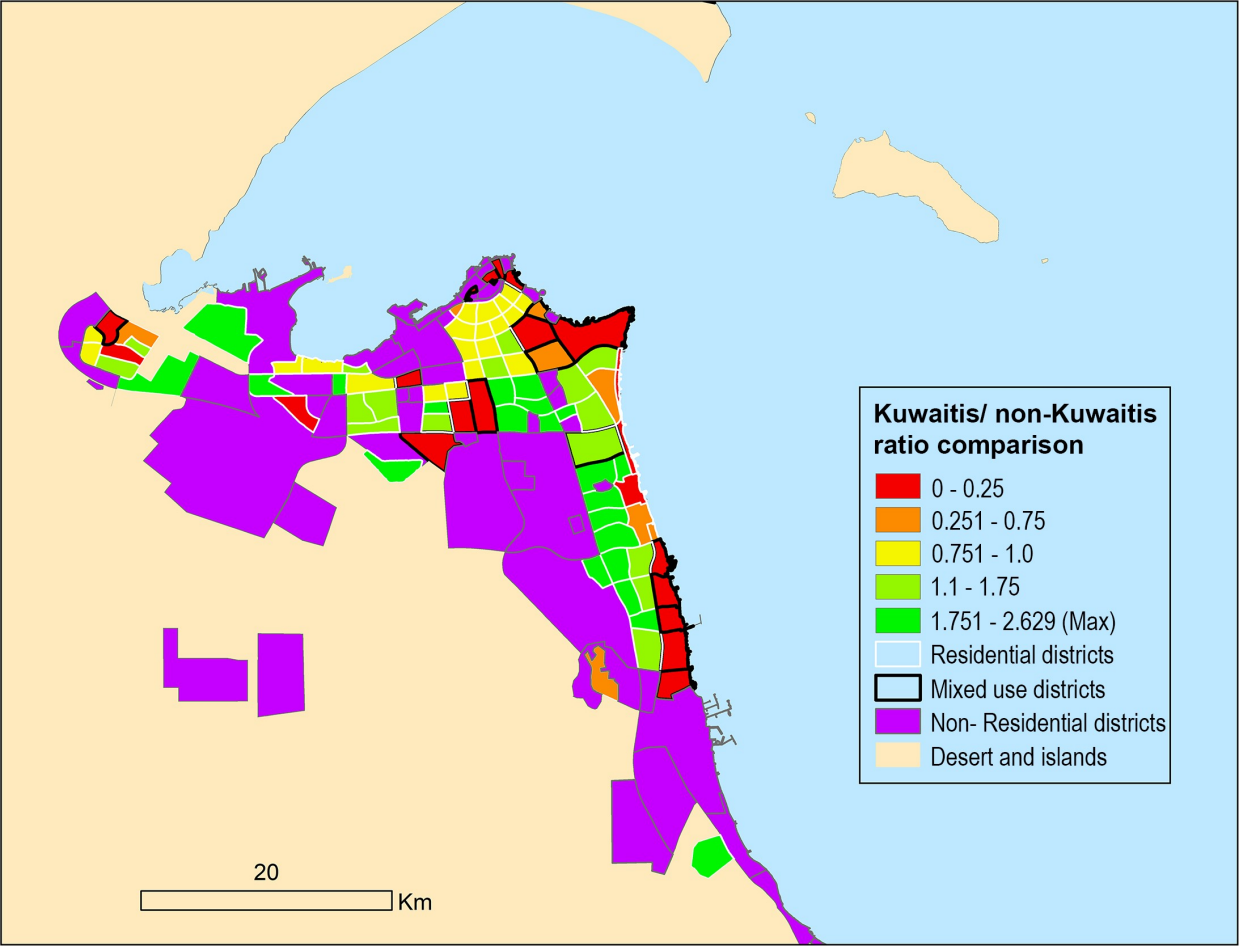
Kuwaitis/non-Kuwaitis distributions in 2015.

The population proportion in mixed districts is 9% Kuwaitis and 91% non-Kuwaitis. In residential districts, non-citizen numbers are still notable, but the majority is living in their employers’ (citizens) dwellings as servants. The high levels of segregation can be seen in Fig 2 for all districts.

### 2.4 Modelling urban development in Kuwait

Due to the relatively small area and population of Kuwait, the high ratio of non-citizens and the existence of a single urban area, the drivers of internal migration are not clearly understood. Furthermore, there is a lack of data to understand the future of internal migration to the new cities, as there has been no significant internal migration occurrence in the past in Kuwait as explained earlier.

A strong pull factor to attract citizens to move to the new cities in Kuwait is supported via government policy by the provision of housing welfare (lands or houses that are almost free) [53]. As of 2017, plans to offer new housing in the new cities have been confirmed [55]. Other possible pull factors attracting residents to new cities include: the creation of job opportunities, the opening of large areas for private sector investments, opening new government offices and new education institutions [56]. Push factors at the origin may include the high cost of living in established areas close to the capital, the long-time of commuting (traffic congestion) and the long waiting list for government supported housing. Pull factors at the origin may include: the desire to live in areas with similar cultural backgrounds, and live close to families and relatives and their current work locations [57].

Cultural diversity is a rather controversial topic with a range of political and social dimensions. In a country with a high number of foreign residents like Kuwait, even internal migration is likely to trigger unpredictable effects and may undermine social solidarity in previously culturally uniform regions. For instance, non-Kuwaitis (South Asians or Egyptians) often concentrate in one district and so do Kuwaitis from the same tribe [51, 58]. Generally speaking, Kuwaiti clusters are found in residential districts and non-Kuwaiti clusters in mixed use districts. Segregation according to nationality may compromise the plan of making Kuwait the financial and trade centre of the region, as it assumes Kuwait’s new cities will be global cities attracting people from around the world. If social segregation between citizens and non-citizens is increased it may also make citizens feel as minority in their country and generally creating a non-integrated society [51, 59].

With the above points, the research considered the following influences for modelling outcomes of new cities:

- Push-pull factors at the origin (existing urban areas) and destination (new cities).
- Resident willingness to move to new cities.
- Effects of nationality segregation.
- New master plan approval by the public.

This paper is part of a larger project that contains two additional scenarios (business as usual scenario: following the historical trends of urban development plus the new master plan scenario: following the government assumptions and perspective) [9, 50, 60]. The new master plan has been evaluated in previous papers from the government perspective without including the residents’ opinions and based on the authorities’ assumptions. The results showed that the plan will provide positive outcomes in terms of housing shortages and traffic congestion if the new cities and the train network can be established on time without delays [50].

## 3. Data and methodology

### 3.1 Resident surveys

#### 3.1.1 Survey design

Due to the lack of data about internal migration and its drivers in Kuwait, it was necessary to conduct a survey for residents to obtain primary data. According to Brown and Robinson [61], data collected directly from residents may be used to identify and understand their preferences and behaviours. The survey questions should be direct, easy to understand and designed in a way that avoids statistical bias. To meet these prerequisites, the survey participants were identified as residents (both Kuwaitis and non-Kuwaitis), above 18 years old and excluding any servant who live in their employers’ home. Furthermore, residents who can understand Arabic or English were selected, as these are the most common languages in Kuwait. Due to the differences between the citizens and non-citizens, the survey participants were separated into two groups (citizens and non-citizens) and two separate question sheets were developed. Table 1 shows the sample and population details and proportions for each survey group.

**Table 1:** Sample and population figures and proportions for each agent group in the simulations.

Agent groups	Age group	Population	% of total population	% of total population (excluding teenagers & servants)	Total in survey	% of survey	% of survey (excluding teenagers & servants)
Kuwaitis	<18	605,864	14%	0	10	1%	0
	18–34	324,504	8%	13%	459	36%	37.5%
	35–49	211,241	5%	8%	257	20%	21%
	50->60	165,996	4%	7%	153	12%	12.5%
	Total =	1,307,605	31%	28%	879	69%	71%
Non-Kuwaitis	<18	484,521	11.5%	0%	27	2%	0
	>18–34	773,173	18%	31%	208	16%	17%
	35–49	722,322	17%	29%	109	8%	9%
	50->60	289,271	7%	12%	41	3%	3%
	Total =	2,269,287	53.5%	72%	385	29%	29%
Servants NK	<18	36	0%	0	0	0%	0
	18–34	292,849	7%	0	11	1%	0
	35–49	313,691	7.5%	0	8	1%	0
	50->60	55,538	1%	0	2	0%	0
	Total =	662,114	15.5%	0	21	2%	0
Total		4,239,006	100%	100%	1,285	100%	100%

The survey invitations were sent via social media such as Twitter, Instagram and WhatsApp, as these applications are the most commonly used in Kuwait [62]. The sample size was calculated according to the methodology proposed by Smith [63]. Specifically, over 2000 invitations were sent, in order to obtain a 95% confidence level, +/- 5% confidence interval (Margin of error) and standard deviation equal to 0.5, as the expected response ratio was 20%. Details about the sample size calculation based on the desired confidence level, standard deviation and expected response ratio can be found in Smith [63]. The invitations were sent via social networks, using the following filters: Kuwait residents, above 18 (excluding servants) that own a social network account. The invitations were sent randomly, but the sample points were stratified according to Kuwait’s age and male/female ratio to reduce sampling bias. This was particularly necessary in regards to age, as younger users were more active in responding via social networks than older users. The sampling data was also separated in citizen/non-citizen groups, according to the participants’ responses about their status.

The survey was online and stayed open for collecting responses for 2 months. 879 responses were collected from Kuwaitis, which represent a 3% of Margin of error, whereas 406 responses were collected from non-Kuwaitis, which represent a 5% of Margin of error [64].

According to Klabunde and Willekens [29], push and pull factors of internal migration are outcomes of people’s decisions in terms of migration. Push factors in the origin location refer to any pressing issues that adversely affect resident lifestyles, such as traffic congestion, unemployment, lack of safety or unaffordable housing. All these may cause locals to consider migration to a different location. On the other hand, pull factors at the origin refer to residents’ ties to their community, families and workplace. Pull factors in the destination, refer to any condition that attracts residents to that location; common examples of pull factors include family ties (if family resides in a different location), employment opportunities, access to high quality education and healthcare and natural environment. Intervening obstacles that may affect internal migration include geographic distance, opportunities present in between the origin and destination or administrative difficulties (for instance obtaining permits) [65].

It should be noted that this research’s surveys were approved as complying with the Australian National Statement on Ethical Conduct in Human Research Regulations and University of Queensland- Human Research Ethics Committee has approved it in 16/2/2016. Besides, eleven questions were developed related to internal migration decisions and locational preferences. These questions relate to the theoretical model of push and pull factors. Question 1 was developed to extract the residents’ decisions about migration. The locations of the new cities were shown to the survey respondents on a map (Fig 1). Questions 2–5 and 11 were designed to understand the main drivers of their decisions (migrate or stay); or in other words the push-pull factors of origin and destination locations. Questions 6 and 10 were designed to obtain the residents’ preferences about district type and future migration locations. Finally, Questions 7–9 served the purpose of collecting inputs and parameters for the migration model. A list of these questions can be seen in Table 2.

**Table 2:** Survey questions for residents.

Q n	For Kuwaitis	For non-Kuwaitis	Question type
1	Kuwait government is planning to develop new cities outside the existing urban area. I am considering moving to these new cities within the next 5–10 years.		Likert scale from 1–5 with 1 being the least favourable (strongly disagree) to 5 being the most favourable (strongly agree).
2	I am considering moving because of financial reasons i.e. lower real estate cost or new employment opportunities. (If answer was Strongly agree or Agree in Question 1).		Likert scale from 1–5 with 1 being the least favourable (strongly disagree) to 5 being the most (strongly agree).
3	I am considering moving because of social or other reasons i.e. to be near to relatives or friends, change of family size or to obtain free dwelling provided from the government. (If answer was Strongly agree or agree in Question 1).	I am considering moving because of social or other reasons i.e. to be near to relatives or friends or change of family size. (If answer was Strongly agree or agree in Question 1).	Likert scale from 1–5 with 10 being the least favourable (strongly disagree) to 5 being the most (strongly agree).
4	I am not considering moving because of financial reasons i.e. living near to work or living cost is reasonable. (If answer was Strongly disagree or disagree in Question 1).		Likert scale from 1–5 with 1 being the least favourable (strongly disagree) to 5 being the most (strongly agree).
5	I am not considering moving because of social or other reasons i.e. to be near to relatives or friends or own a house/ apartment. (If answer was Strongly disagree or disagree in Question 1).		Likert scale from 1–5 with 1 being the least favourable (strongly disagree) to 5 being the most (strongly agree).
6	*Preference for what region of Kuwait*: I prefer to reside in residential districts (Fiha, Surra or Audiliya) rather than mixed districts (Salmiya, Hawalli or Khaitan).		Likert scale from 1–5 with 1 being the least favourable (strongly disagree) to 5 being the most (strongly agree).
7	Household size		Choosing number from 1–20.
8	Number of servants residing in my household’s premises		Choosing number from 0–10.
9	*Preference for locational features*: Please order the following criteria/ elements based on your demands and wishes: - Closeness to government services such as ministries and organisations. - Closeness to public services such as shopping malls, hospitals, universities and others. - Closeness to the sea/ beaches. - Closeness to the existing urban area. - Closeness to airports. - Closeness to public transportation (bus or train networks). - Low cost of dwellings (purchase or rent).		Ordering (From 1 = Most important to 7 = Less important).
10	Please choose your first preference for settlement location based on your demands and wishes: - Stay inside the existing urban area. - New city in the North side of Kuwait. - New city in the Middle side of Kuwait. - New city in the West side of Kuwait. - New city in the South side of Kuwait.		Single choice answer.
11	Which of these factors may affect your decision of not moving from the existing urban area and make you change your answer to a new city? (If in question 10, answer was a.) - High pressure on land and property values in the existing urban area. - Housing shortages in the existing urban area. - Very long commuting times in the existing urban area. - High rates of accidents in the existing urban area. - All needed public services provided in the new city. - Open a branch of your job in the new city. - New modern train network established. - Larger house sizes in the new city.	Which of these factors may affect your decision of not moving from the existing urban area and make you change your answer to a new city? (If in question 10, answer was a.) - High pressure on land and property values in the existing urban area. - Housing shortages in the existing urban area. - Very long commuting times in the existing urban area. - High rates of accidents in the existing urban area. - All needed public services provided in the new city. - Open a branch of your job in the new city. - New modern train network established.	Multi-choice answers.

#### 3.1.2 Data processing

The survey data was firstly disaggregated in different age and nationality categories. A separate category for servants was implemented, as they live in their employers’ dwellings and do not have the ability to make any decision for migration.

The agent groups formed and used in the ABM were:

1. Kuwaitis- teenagers (<18).
2. Kuwaitis- young adults (18–34).
3. Kuwaitis- middle aged (35–49).
4. Kuwaitis- seniors (>50).
5. Non-Kuwaitis- teenagers (<18).
6. Non-Kuwaitis- young adults (18–34).
7. Non-Kuwaitis- middle aged (35–49).
8. Non-Kuwaitis- seniors (50->60).
9. Servants (non-Kuwaitis).

These population categories were assigned to actual agents in the ABM, the details of which are presented in section 3.2.1. A possible sampling problem of bias may arise as it is expected that more young adults will be attracted to participate rather than seniors. To make sure this statistical bias is avoided, a correction technique called post-stratification weighting adjustment [66] was used to ensure that the sampling is stratified to the population demographics. The adjustment factors were gender, age and nationality. To determine any significant differences between citizens and non-citizens in their responses, a single way ANOVA test was utilised. It should be noted that while teenagers and servants constitute separate agent groups in the ABM simulations, they do not have independence in deciding whether or where to move, as they have to follow their parents or employers respectively.

Table 3 provides the summary of the respondent profiles, including the total numbers of responses for each group, and several demographic characteristics.

**Table 3:** Survey responds information.

Classification	Nationality	
	Citizens (Kuwaitis)	Non-citizens (Non-Kuwaitis)
**Number**	879	406
**Gender**	Male: 52% Female: 48%	Male: 71% Female: 29%
**Age**	<18–34: 53% 35–49: 29% 50->60: 18%	<18–34: 60% 35–49: 29% 50->60: 11%
**Employment status**	Student: 16% Employed: 64% Unemployed: 3% Retired: 13% Other: 4%	Student: 27% Employed: 59% Unemployed: 4.5% Retired: 0.5% Servants: 5% Other: 4%
**Marital status**	Never married: 30% Married: 63% Divorced: 6% Widower: 1%	Never married: 38.5% Married: 55.5% Divorced: 4% Widower: 2%
**Educational background**	Less than bachelor degree: 25% Bachelor: 61% Post graduate degree: 14%	Less than bachelor degree: 36% Bachelor: 53% Post graduate degree: 11%
**Residential status**	Own house: 28% Rented house: 7% Own apartment: 2% Rented apartment: 18% Living in parents’ house: 45%	Own house: 10% Rented house: 19% Own apartment: 3% Rented apartment: 67% Living in Kuwaitis house as servant: 1%

Table 4 summarizes the differences and similarities between Kuwaitis and non-Kuwaitis based on the ANOVA test. For more details for the survey responds, see -supplementary materials (S1 File).

**Table 4:** Differences and similarities between Kuwaitis and non-Kuwaitis in their responds.

Q n	Is there a significant difference between citizens and non-citizen responses? (one way ANOVA)	Interpretation
1	There were no statistically significant differences between group means (*F(1*,*1232)* = 0.088, *p* = 0.767 > 0.05).	Nationality does not affect intention to move to new cities.
2	There was a statistically significant difference between group means (*F(1*, 456) = 13.117, *p* = 0.000 < 0.05).	Economic pull factors are more important to non-citizens.
3	There was a statistically significant difference between group means (*F(1*, 456) = 11.218, *p* = 0.001 < 0.05).	Social and other pull factors are more important to citizens.
4	There were no statistically significant differences between group means (*F(1*, 279) = 3.005, *p* = 0.084 > 0.05).	Same economic reasoning for staying for both groups.
5	There were no statistically significant differences between group means (*F(1*, 279) = 1.059, *p* = 0.304 > 0.05).	Same social and other reasons for staying for both groups.
6	There was a statistically significant difference between group means (*F(1*, 1008) = 594.208, *p* = 0.000 < 0.05).	Kuwaitis prefer residential districts, whereas non-Kuwaitis prefer mixed districts.
7	Not applied	Not applied
8	Not applied	Not applied
9	Not applied	The ranking of criteria is different between citizens and non-citizens.
10	There was a statistically significant difference between group means (*F(1*, 916) = 4.177, *p* = 0.041 < 0.05).	Preferred locations of settlement are different between citizens and non-citizens.
11	Not applied	The same push and pull factors affect both groups’ decision of moving.

**Fig 3:**
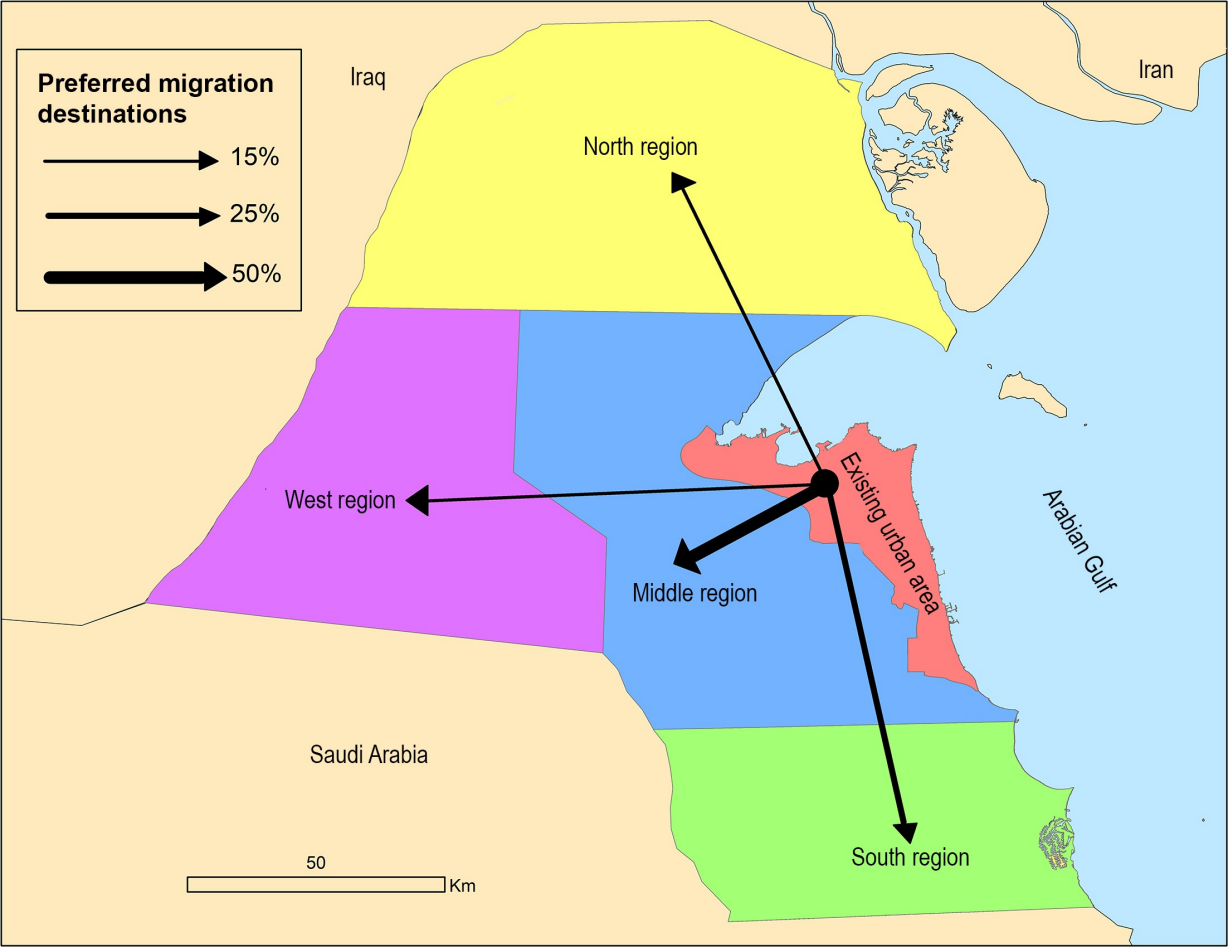
Migration destinations preferences from survey (Question 10).

Fig 3 shows the preferred migration destinations according to the survey results (Question 10) from the existing urban area to new regions.

### 3.2 Model and scenario design

#### 3.2.1 Model design and concept

There are several tools available for ABM that describe simulation models in terms of agents, environment, scheduling, interacting and rules and behaviours [67–69]. Integrating agent interactions and the spatial movement of these agents was challenging with available ABM tools so the model was implemented in a programming language, namely Python, which is integrated with GIS software. The implemented model follows the same procedures and simulation control as supported in ABM tools, but allows a higher level of coupling with spatial data as provided by GIS. There were two spatial scales in the model: national and district. The simulations run in 5-yearly intervals from 2015 to 2050, for a total of 7 time steps. The model assumed that population and land use distribution are going to evolve according to three different scenarios as mentioned earlier. Table 5 explains the details of these scenarios.

**Table 5:** Scenarios characteristics.

Scenario	Characteristics			
	Modelling type	Establishing new cities	Migration	Nationality segregation
1	Top-down	Based on government authorities plans	Based on government assumptions and expectations	Based on government district spilt policy and historical trends.
2	Bottom-up	Based on resident preferences, demands and choices as extracted from the surveys.		Based on resident preferred district type as extracted from the surveys.
3				All new districts are similar and there is no spilt policy or preferred resident district type.

The model involves two types of agents:

- Decision agents (government planning authorities).
- Resident agents (mobile agents able to migrate from existing urban areas to the new cities).

The resident agents are divided into two categories (citizens and non-citizens). These categories were further classified into 9 classes, according to the factors discussed in 3.1.2. Table 6 shows the agents groups.

**Table 6:** Model’s agents.

Agent	Classes	Actions	Behaviours
Government authorities	-	Establish the new cities in scenario #1.	Responsible for urban planning and establishing new cities and infrastructure.
Citizens (Kuwaitis)	Teenagers	Stay in current residence or migrate to new cities with their parents.	Have no ability to make any decision independently.
	Young Adults	Have the ability to change the order of establishing cities based on their preferences and needs (in scenarios #2 and #3). Choose to stay in current residence or migrate to new cities.	Prefer to settle in residential districts. High chances of migration.
	Middle aged		Prefer to settle in residential districts. Average chances of migration.
	Seniors		Prefer to settle in residential districts. Low chances of migration.
Non-citizens (Non-Kuwaitis)	Teenagers	Stay in current residence or migrate to new cities with their parents.	Have no ability to make any decision independently.
	Young Adults	Have the ability to change the order of establishing cities based on their preferences and needs (in scenarios #2 and #3). Choose to stay in current residence or migrate to new cities.	Prefer to settle in mixed use districts. Low chances of migration.
	Middle aged		Prefer to settle in mixed use districts. Average chances of migration.
	Seniors		Prefer to settle in mixed use districts. High chances of migration.
	Servants	Stay in current residence or migrate to new cities with their employers.	Have no ability to make any decision independently.

**Fig 4:**
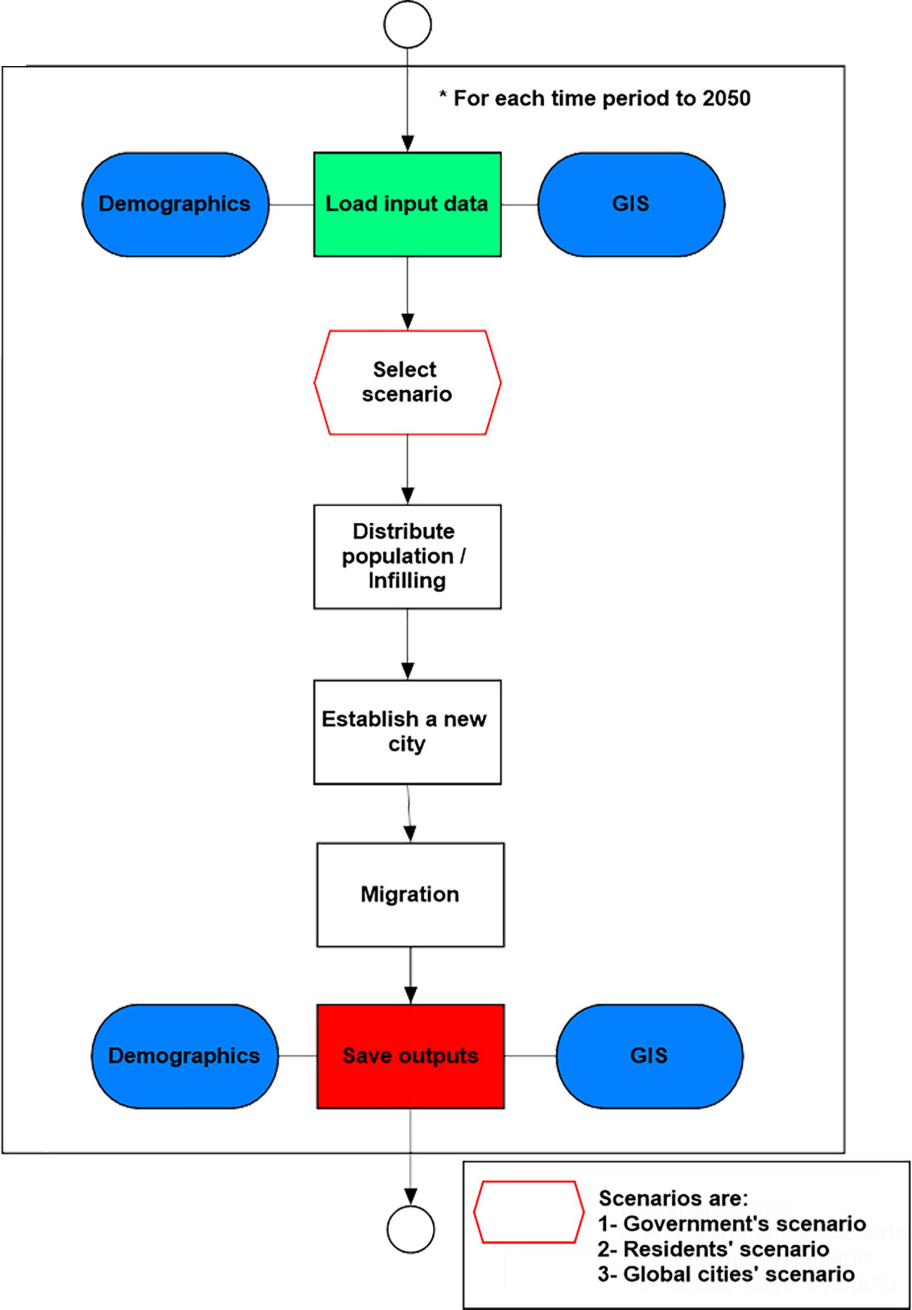
The model flowchart.

Each of the nine resident agents was represented as resident agent locations (point features in GIS) for spatial visualization of migration in the model (each point = 100 persons). The environment was represented as a polygon feature class that includes the land use as districts. Fig 4 shows the model flowchart.

The decision-making process in modelling typically involves two steps: firstly, an assessment of the choices about migration and secondly the transformation of the assessment results into an action [29]. However, in this simulation and as data was directly collected from the residents, the decision-making process includes three steps.

The first step is about establishing or not establishing new cities according to the number of people willing to move, as derived from the surveys. The threshold percentage of residents willing to move, above which new cities are established is 50% of the new city establishment capacity [9]. The second step applies the migration decision as collected from the responses to Question 1. Finally, in the third step the resident migration actions are carried out to any new location based on their preferences as collected from Questions 9 and 10. These actions occur under a few important conditions:

- There are vacant lots in the district.
- Migration may be only towards the new cities (old districts can be occupied by residents in infilling step).
- The residents that would want to migrate to the new city exceed the aforementioned threshold percentage.

Certain assumptions had to be made during the model design, due to lack of data and to promote realistic representation of the urban development in Kuwait. These assumptions are summarized in Table 7.

**Table 7:** Model main assumptions.

Assumption	Reason	Effects
Employment opportunities, public services, housing and infrastructure distribution are spatially uniform in new cities.	Stated in Kuwait municipality’s master plan and Public Authority of Housing Welfare’s dwellings provision plan.	There are no resident preferences based on these parameters.
Distributing resident age classes and servants in existing suburbs was based on averages.	Lack of data.	All existing districts have the same distribution of age groups and servants.
Current resident preferences will be applied in all future time steps.	Lack of future data.	Each time step will has the same preferred locations and district type and migration ratio.
New cities will be initially filled 25% of its maximum capacity via internal migration.	According to historical data new cities need more than 5 years to be occupied by residents.	New city will initially house only a few residents and will be filled via infilling in the following time steps.
No financial and political changes that may increase instability will occur.	Outside of scope of this paper.	Stable conditions for urban development practices.
The maximum capacity of existing districts will be the same as 2015.	Lack of data.	Existing districts will not host more residents than in 2015 (could be less).
Household size for Kuwaitis is 7 (assuming 2 parents, 3 kids and 2 servants)	Based on the survey averages.	This will affect the establishing new cities action.
Household size for non-Kuwaitis is 5 (assuming 2 parents, 2 kids and 1 servant)	Based on the survey averages.	This will affect the establishing new cities action.

#### 3.2.2 Model inputs

The model uses two types of spatial and population data to simulate population migration: 1) locational suitability parameters and 2) model demographic variables.

The locational suitability parameters were calculated with the *Near* tool in ArcGIS and according to existing and future infrastructure. Costs were calculated with the help of real estate annual reports [70]. The suitability weights were determined by using the Analytic Hierarchy Process (AHP) [71]; this statistical method involves transforming the resident survey responses from Question 9 into weights according to the number of options (6 in this case) and their rank. For instance, the highest ranked option was awarded a weight of 35%, the second ranked option a weight of 24% and so on. Further information about the AHP method can be found in relevant literature [71]. The weights affect the decision of establishing new cities in scenarios #2 and #3 and affect the distribution of residents in all scenarios based on their preferences. The ranked responses from both groups as determined from the survey responses, as well as the suitability weights as calculated can be seen in Table 8 (top rows presents parameters ranking similarity and lower rows shows the differences).

**Table 8:** Locational suitability parameter rankings and weights.

Parameter Ranking	Kuwaitis	Non- Kuwaitis	Weight
1	Lower cost of dwellings (purchase or rent).	Lower cost of dwellings (purchase or rent).	35%
4	Closeness to government services.	Closeness to government services.	10.5%
7	Closeness to airports.	Closeness to airports.	3%
2	Closeness to the existing urban area.	Closeness to public services such as shopping malls, hospitals, universities.	24%
3	Closeness to public services such as shopping malls, hospitals, universities.	Closeness to the existing urban area.	16%
5	Closeness to the sea/ beaches	Closeness to public transportation (bus or train).	7%
6	Closeness to public transportation (bus or train).	Closeness to the sea/ beaches	4.5%

The model’s demographic input variables include future demographic data with aggregate projections from 2015–2050 by nationality and age group [72] and allowed predictions for international migration, death rates, birth rates and nationalization (the process of obtaining Kuwait citizenship). This data may be modified over time. Variables collected from the surveys, but do not change over time, were classified spatially and can be seen in Table 9.

**Table 9:** Collected input variables.

Variables		Residents’ agents					
		Citizens			Non-citizens		
		Young Adults	Middle aged	Seniors	Young Adults	Middle aged	Seniors
Migration Desire %		40%	35%	35%	44%	36%	45%
Preferred region	Existing urban area	42%	41%	53.5%	54%	53%	49%
	North region	9%	11%	2.5%	6%	7%	2.5%
	Middle region	36%	32.5%	31%	25%	20%	29%
	West region	5.5%	4%	8%	9%	7%	0%
	South region	7.5%	11.5%	5%	6%	13%	19.5%
Preferred district type	Residential	92%	91%	94%	36.5%	30%	28%
	Mixed	8%	9%	6%	63.5%	70%	72%
Household size		7			5		

#### 3.2.3 Model algorithm

**Fig 5:**
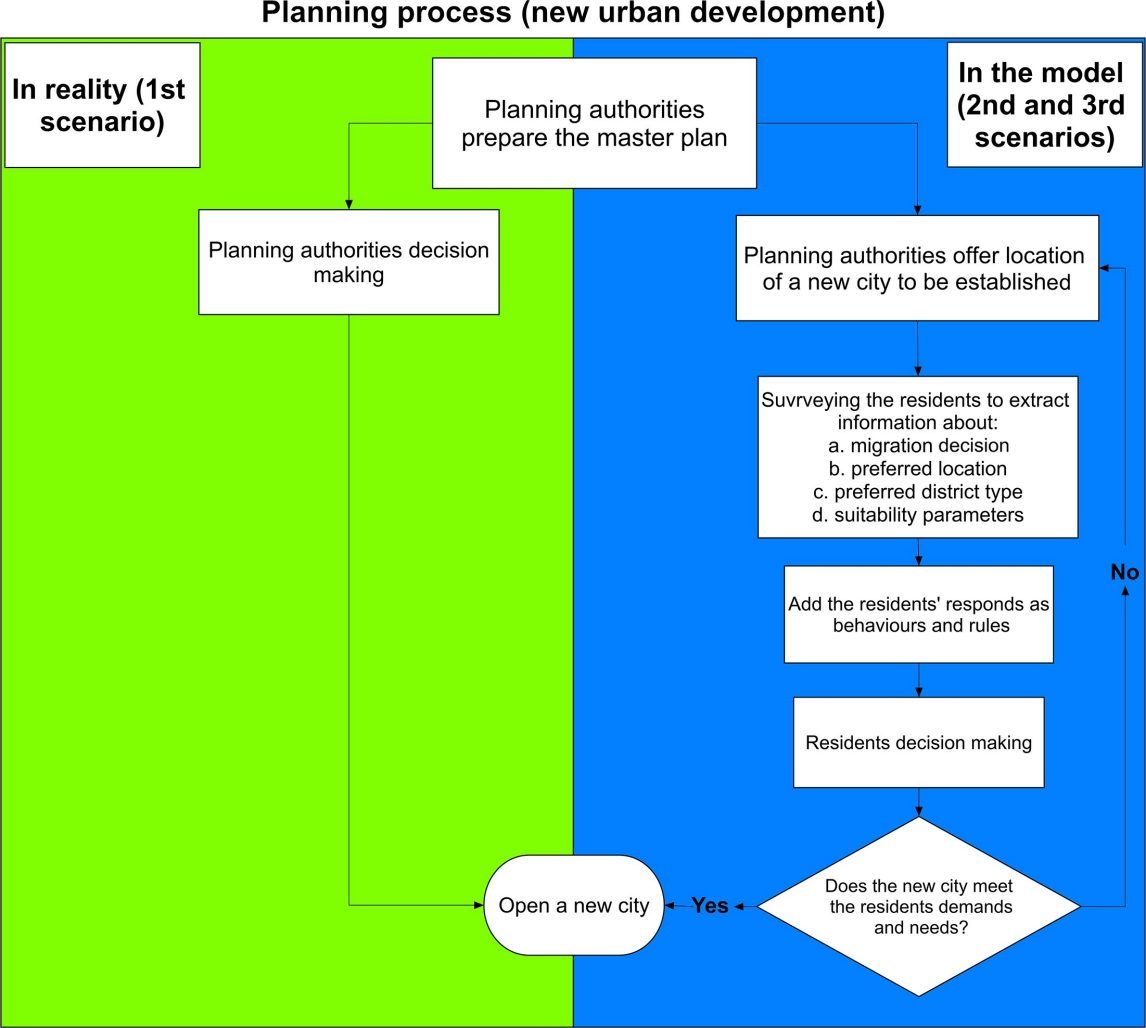
Framework of involving residents in future plans model.

The algorithm steps for the simulations (see Fig 4) are as follows:

1. Initialization: Firstly, the spatial environment is created in GIS, initialising the default parameter values and input variables, loading population projections and initialising the model schedule to begin in 2015.
2. Preparation and calculation: This step includes calculations of the suitability weights for the new cities and the districts inside new cities and calculations for the threshold for opening the new cities.
3. Infilling: This step allocates new residents to old districts (including new open districts if any available) with available housing capacity. Residents are distributed based on their agent class and in way that is aligned with the age distribution averages. The numbers of residents are converted into resident agent locations points in the map (i.e. for each residential classes as in Table 6). For scenarios #2 and #3, new cities will opened only if the survey participants state that such new cities can meet their needs and expectations. Fig 5 shows the difference in algorithm steps when deciding whether to open a new city or not at each time step.
4. Selecting the scenario: The user may select which scenario will be simulated at this stage.
5. Establishing a new city: for scenario #1 cities will be established according to the master plan. For scenarios #2 and #3, suitability weights, numbers and desires of residents, as well as new city capacity and threshold percentage will be taken into account.
6. Migration: This will be done by moving the resident agents from the old urban area to the selected city. The initial migration will fill 25% of the new city’s capacity. Agents will be added to the new districts based on suitability weights. In addition, residents under 18 years old and servants will follow their parents/employers according to average household size. Finally, the residents that moved to new cities will be removed from the existing urban area.
7. Segregation distribution: This step will be different depending on the scenario as seen in Table 10.
8. Calculation of nationality segregation outcome: the ratio of Kuwaitis to Non-Kuwaitis can be calculated at this step as:

**Table 10:** Segregation distribution method based on scenarios.

Scenario	Segregation Distribution
1	As per the current distribution ratio averages.
2	According to survey responses (Question 6) (resident preference of district type).
3	Uniformly to all new districts (all being mixed type).

$$Nationality\;segregation\;ratio=\frac{Total\;Kuwaitis\;in\;mixed\;districts}{Total\;non-Kuwaitis\;in\;mixed\;district}$$

At the end of each time step output maps, tables and figures are produced to show the simulation results for the population distribution and migration patterns, new city development stages and nationality segregation levels. Details about the ABM in this paper follow the ODD (Overview, Design concepts, Details) protocol can be seen in the supplementary materials (S2 File) [73].

## 4. Results

**Fig 6:**
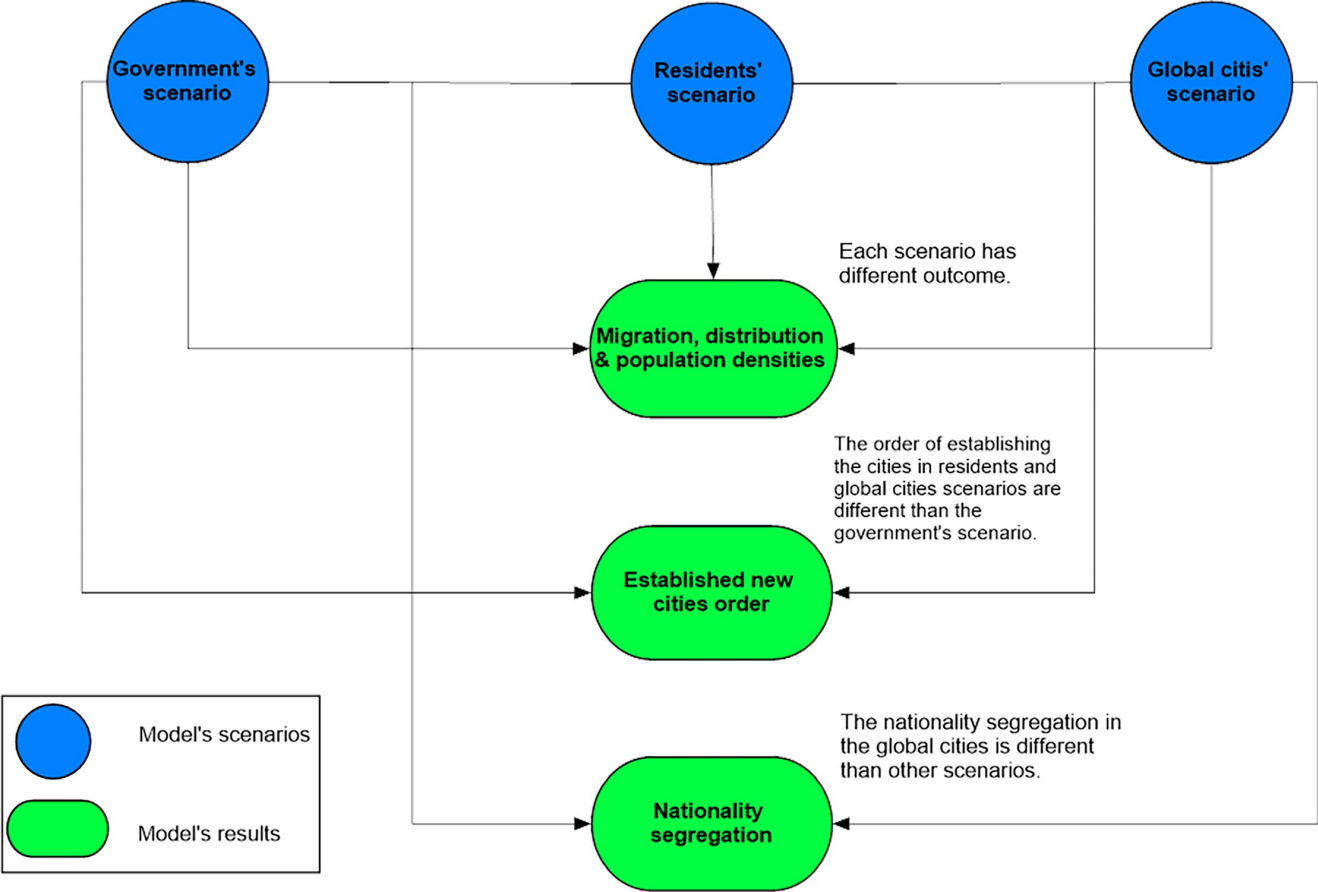
Model’s results differentiations.

Results shown in this section are the average or mode values out of 35 runs of the model. Fig 6 summarizes the main outputs for each model scenario.

The results section will present the model’s results for the different scenarios in three different outcomes: a) future migration, distribution and densities in 2050, b) the order of establishing the new cities and c) the future nationality segregation levels. Therefore, Table 11 shows the predictions for density and demographics of 2050 for each scenario compared to the current situation (2015).

**Table 11:** Scenarios predictions of demographics and distributions.

Scenario	Preferred primary residence regions						
	Citizens			Non-citizens			
	Young Adults	Middle aged	Seniors	Young Adults	Middle aged	Seniors	
**Current (2015)**	Existing urban area			Existing urban area			
**#1** **(2050)**	1.Existing urban area 2. Middle 3. South	1. Existing urban area 2. Middle 3. North		1.Existing urban area 2. Middle 3. South	1. Existing urban area 2. North 3. Middle		1.Existing urban area 2. Middle 3. South
**#2** **(2050)**	1. Existing urban area 2. Middle 3. North	1.Existing urban area 2. North 3. Middle		1.Existing urban area 2. Middle 3. North	1. Existing urban area 2. North 3. South		1.Existing urban area 2. Middle 3. North
**#3** **(2050)**	1.Existing urban area 2. North 3. Middle	1. Existing urban area 2. Middle 3. North		1.Existing urban area 2. Middle 3. North	1. Existing urban area 2. North 3. Middle		1.Existing urban area 2. Middle 3. North

According to the survey responses, the South region ranked higher in preference, however simulation results showed that the North region will host more residents. This is due to the suitability weights of the North region that are higher than the South region cities and the fact that North region cities have higher capacities and hence may host more residents. The West region does not appear to be in the top 3 regions for any group, due to its high distance to the current urban area and the coast. The Middle region is the second most preferred in most cases, most likely due to its closeness to the existing urban area and existing public services. Finally, the existing urban area is still predicted to be the most highly preferred region for all the residents even by 2050. Based on the future resident distribution in Table 11 possible reasons behind these outcomes are:

1. Survey data or simulation such as:
  - The simulation period is not long enough for the new city pull factors to reach high level of desirability.
  - The residents are concerned about delays in new cities projects
  - The residents prefer to be stay close to their current work places and current public services.
2. Real world reasons such as:
  - Senior citizens who own a dwelling do not desire to move.
  - Young citizens who live in their parents’ dwellings are content with their current situation.
  - Push factors of the existing urban area affect only young and middle aged residents.

**Fig 7:**
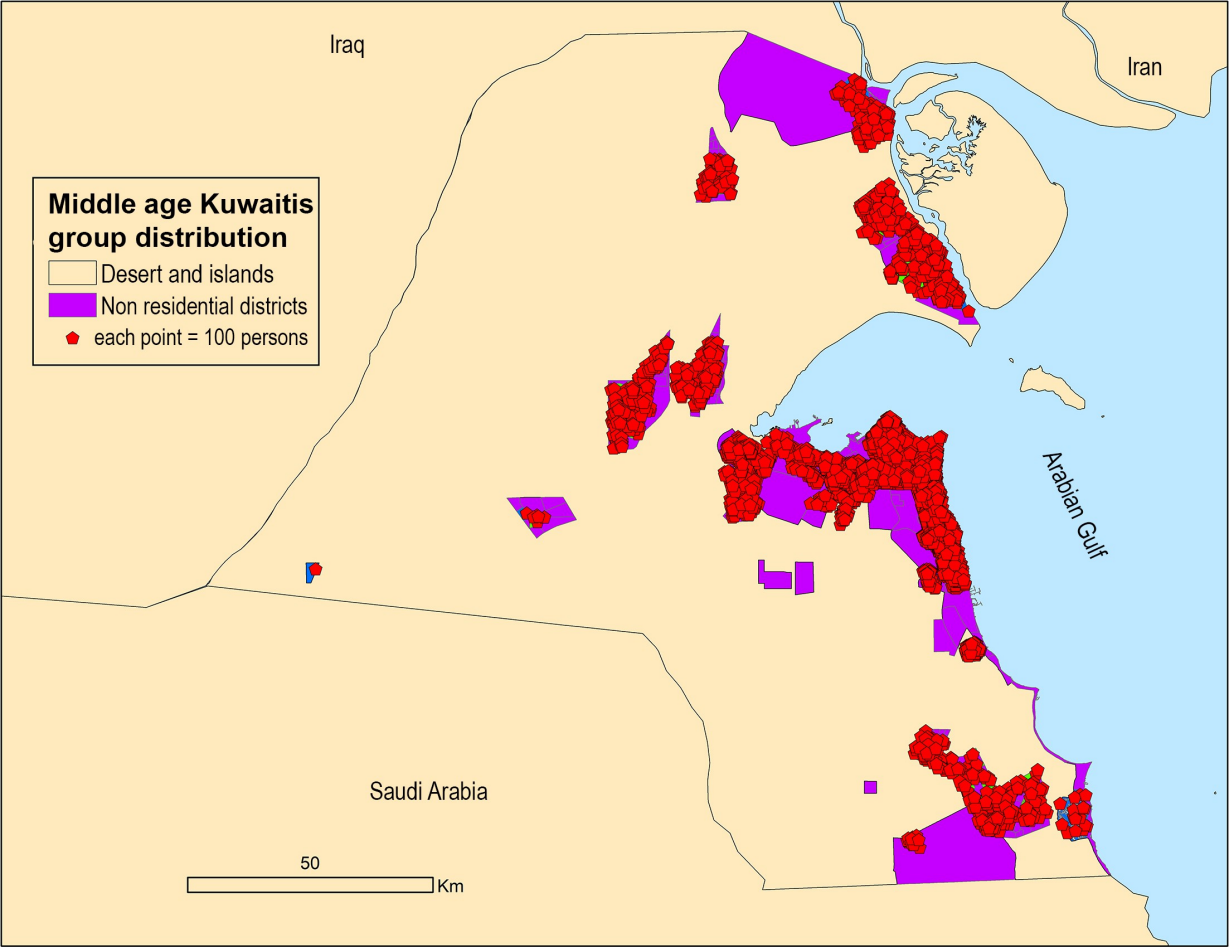
Agents group distributions in 2050.

Although the population is expected to be higher in 2050 compared to 2015, the urban density will drop by more than 50%. Fig 7 shows a typical output map for middled age citizen distribution in 2050.

**Fig 8:**
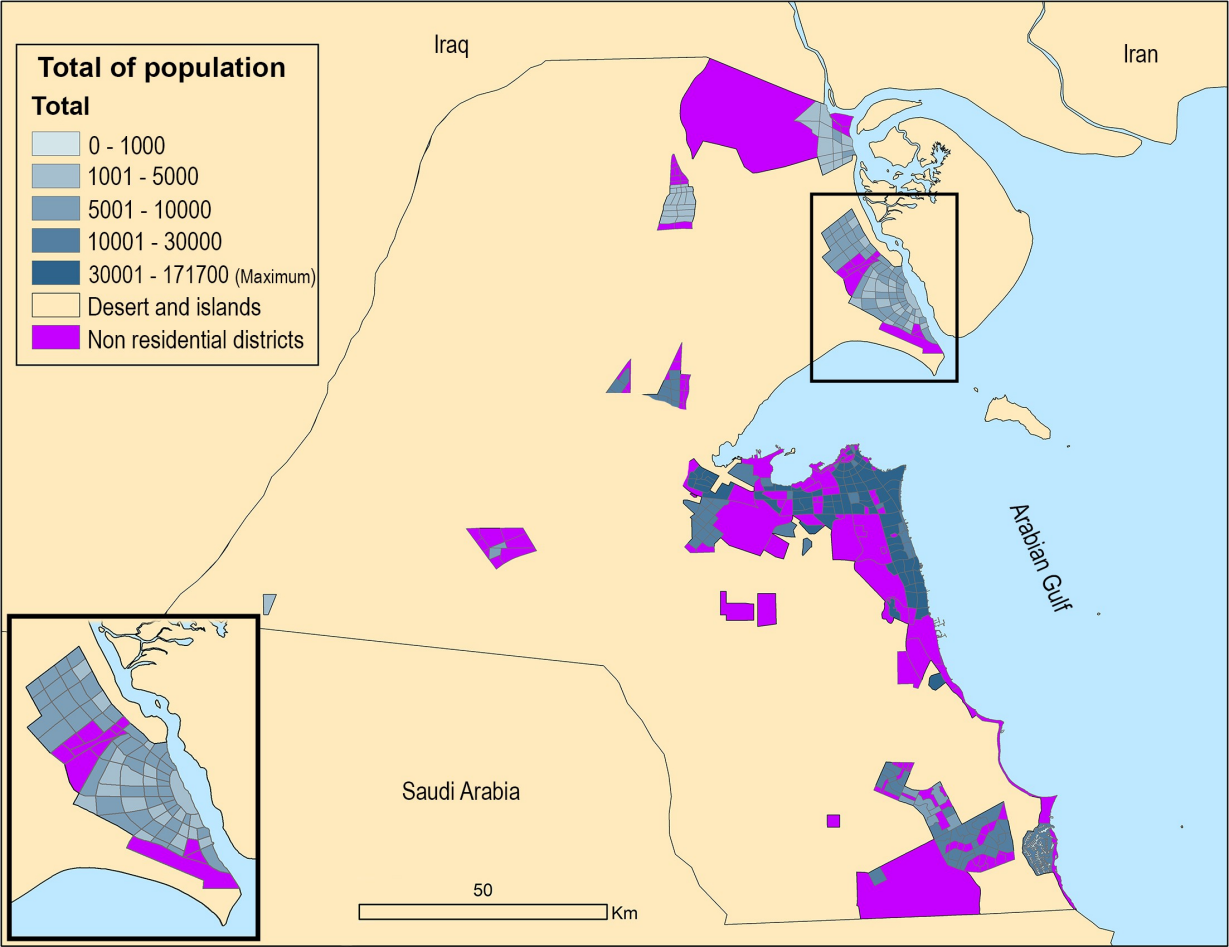
1^st^ scenario population distribution.

**Fig 9:**
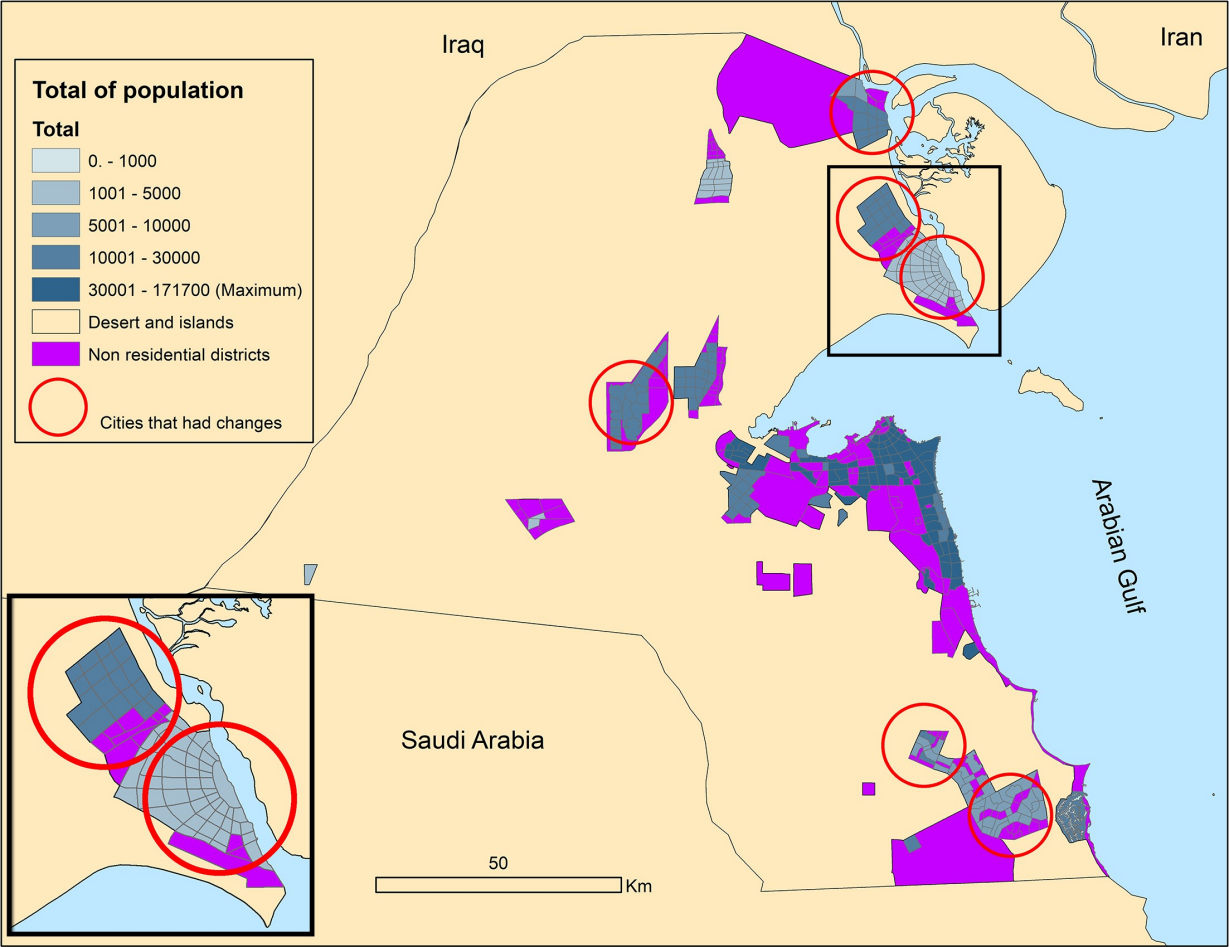
2^nd^ and 3^rd^ scenarios population distribution with differences to 1^st^ scenario.

Figs 8 and 9 show the population distributions maps and highlight any differences between the scenarios.

Table 12 shows the order of establishing the new cities according to the 3 scenarios.

**Table 12:** Establishing new cities order based on scenarios.

City	Region	Establishing year according to scenarios	
		1^st^ scenario	2^nd^ and 3^rd^ scenarios
1	South	2020	2030
2	Middle	2020	2025
3	South	2025	2035
4	Middle	2025	2020
5	South	2030	2035
6	Middle	2030	2025
7	North	2035	2025
8	North	2035	2040
9	North	2040	2040
10	West	2040	2045
11	West	2045	2045
12	North	2045	2030

It can be seen that the order based on citizen responses is different than the master plan proposed order, which highlights the importance of participatory planning.

**Fig 10:**
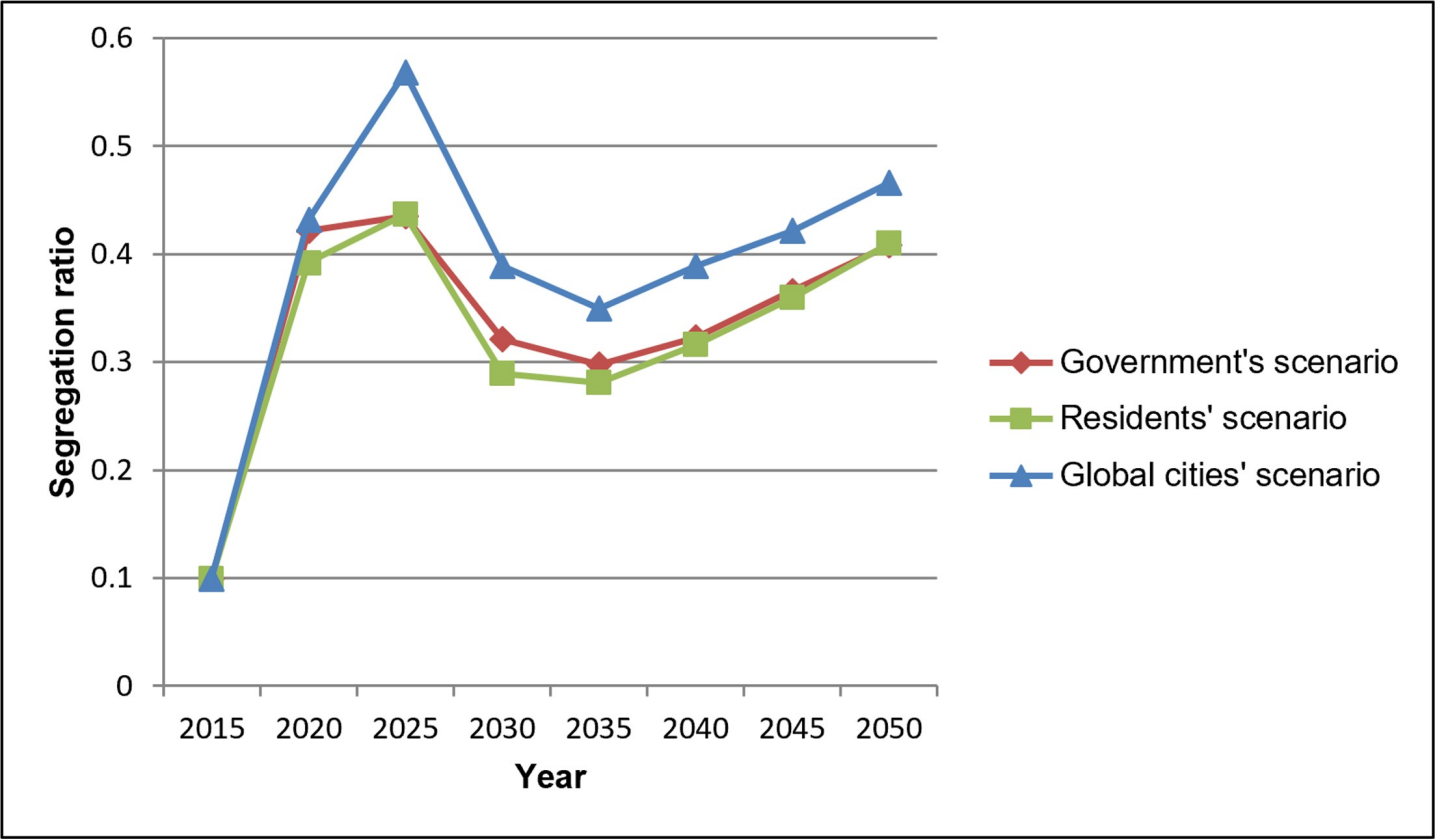
Nationality segregation ratios in different scenarios.

The diagram in Fig 10 shows the nationality segregation ratios for each time step in each scenario simulation (ratio of 1 means no segregation between residents).

**Fig 11:**
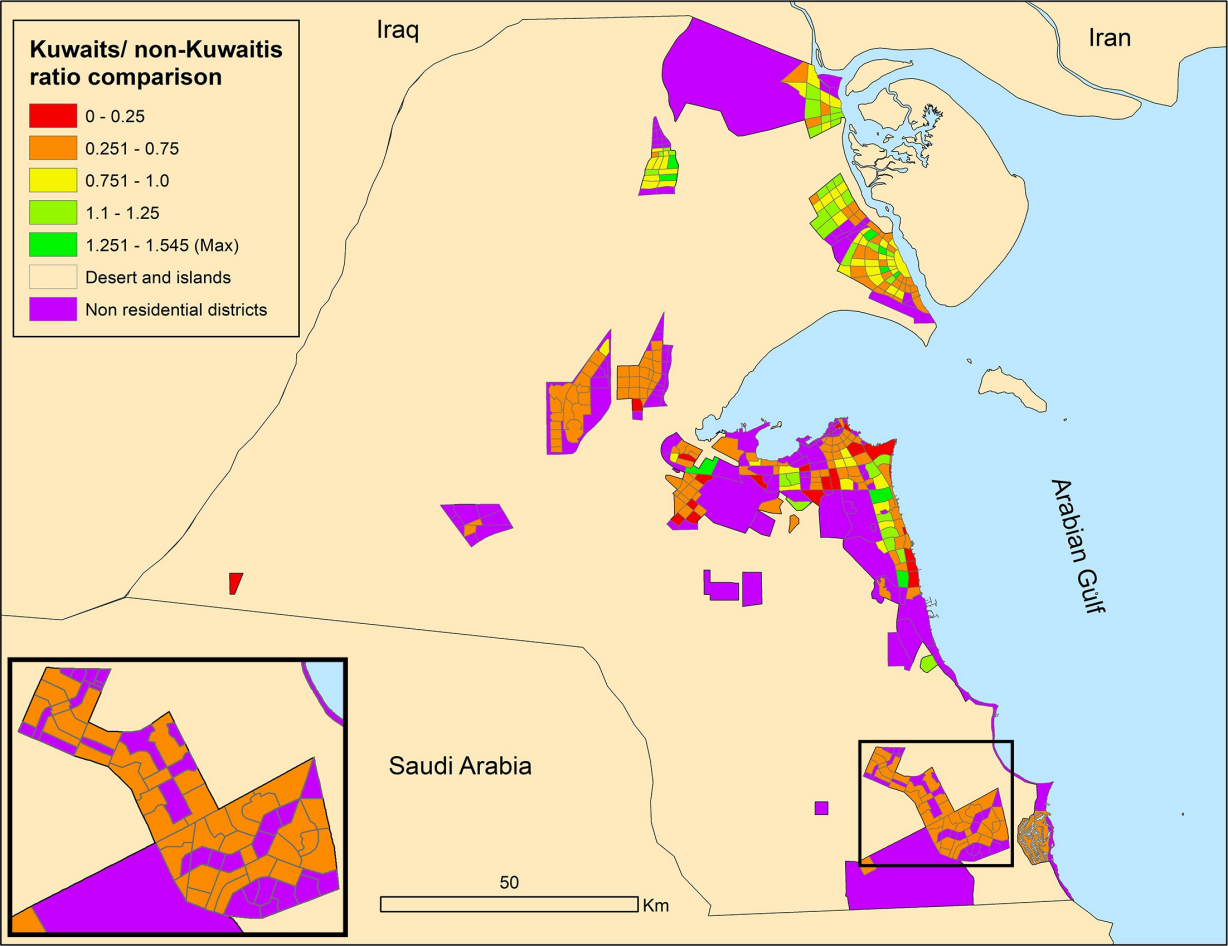
1^st^ scenario segregation outcome map.

**Fig 12:**
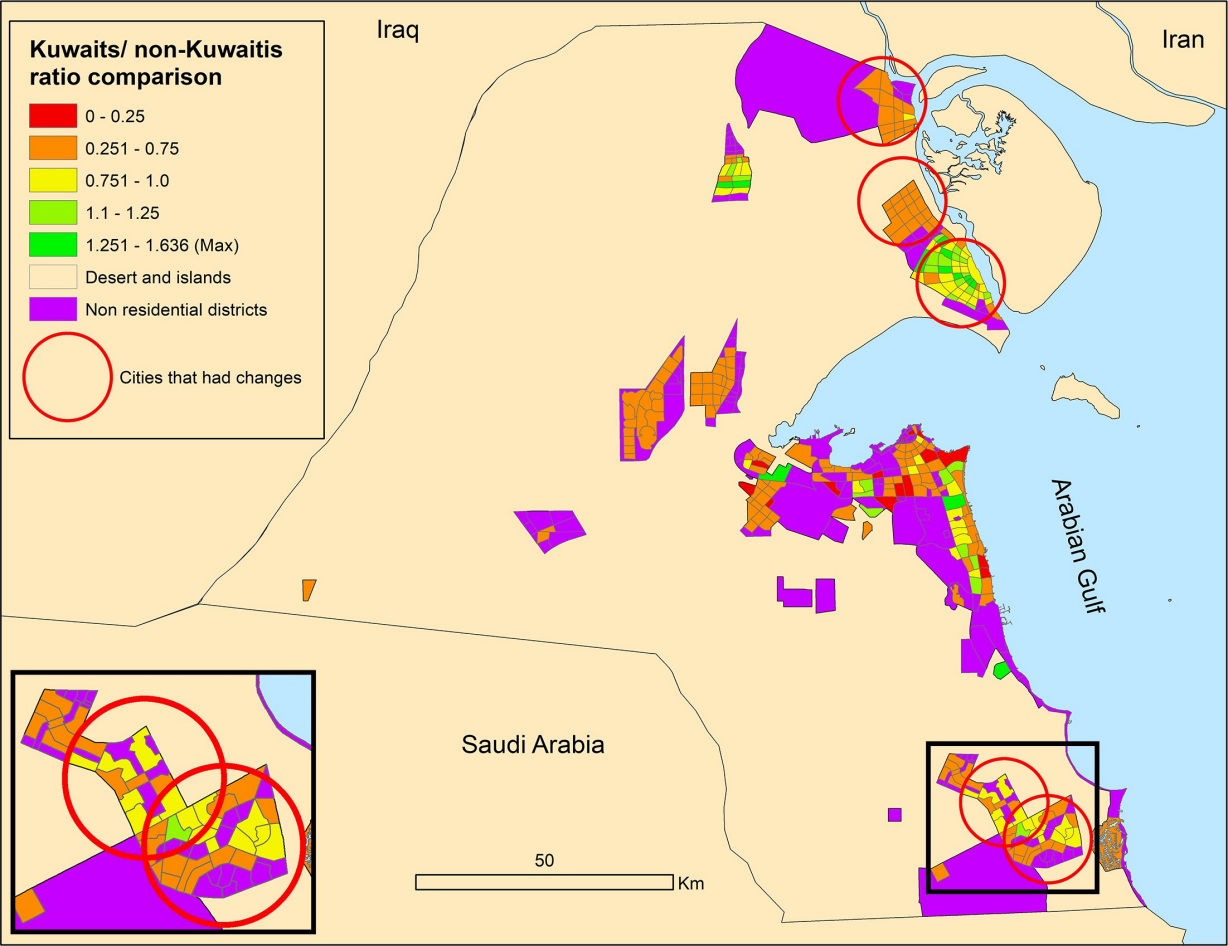
2^nd^ and 3^rd^ scenarios segregation outcome map with differences to 1^st^ scenario.

In all scenarios the segregation ratios will be lower compared to the current situation and the Global cities scenario will present the lowest segregation ratio between residents. Figs 11 and 12 show the nationality segregation ratio maps, with 1 representing equal distribution of Kuwaitis and non-Kuwaitis.

In all scenarios the segregation levels will be lower across all new regions compared to the existing urban area. This trend is mainly due to the allocation of residents being only in the new cities.

## 5. Discussion and conclusions

Three scenarios were developed and simulated with Agent Based Modelling (ABM) as a purely empirical model, to address a series of research questions in relation to the nationality segregation levels and the desirability of Kuwait’s new cities in terms of internal migration by 2050. The simulation outcomes signify the importance of public participation in urban planning. While this is not considered by the government in Kuwait or countries with similar conditions at present, the model of this paper may form a basis for integrating resident opinions in the design and evaluation of construction projects in the future.

The online survey used in this research succeeded as a participatory planning approach to involve residents in land use decisions, and the results used in an agent-based migration model to assess outcomes of future plans. The survey allowed extracting residents’ choices, demands, needs and preferences. These were in turn used in the model as inputs and behaviours for the agents. The results showed that the new master plan of Kuwait will most likely not create ghost cities, as there is interest and sufficient number of residents willing to move to the planned new cities.

From the survey it was clear that the main drivers of internal migration were related to housing and new employment opportunities. However, it was also noted that there were some differences between citizens and non-citizens in their needs and preferences. The results of the survey and the outcomes of the model suggested that there were no significant differences in terms of deciding whether to migrate or not. However, the preferred new settlement regions and district types were different between the two groups. In addition, economic and social reasons behind the decision of not moving to the new cities (pull factors at origin) are similar; whereas, the pull factors (at destination) that new cities offer to attract residents are different. This was the main factor used to distinguish agent groups based upon citizens and non-citizens in the model.

It was no surprise that young and middle aged Kuwaitis had the highest desire to migrate to the new cities, as these age groups are the primary recipients of the welfare housing. However, it was surprising that non-Kuwaiti seniors are the keenest on migration. This could be explained because many non-Kuwaitis are nearing retirement age and they may have to migrate back to their home countries if they are not employed. Non-citizens typically work under contracts of various lengths, so retirement age may actually be relatively early. The new cities may offer new employment opportunities and entice them to stay in Kuwait instead of terminating their stay in the country after their contract expires. This is a particularly attractive prospect to most non-Kuwaitis, as leaving Kuwait would mean they lose their high salaries and social benefits (healthcare, security) relative to their home countries.

Furthermore, this illustrates how the push-pull drivers behind migration are different between citizens and non-citizens in Kuwait. The major pull factor at the destination for Kuwaitis and for non-Kuwaitis is housing, and employment opportunities respectively. Push factors at the origin were found to be similar: high pressure on land and property values and long commuting times in the existing urban area.

The results showed that the nationality segregation levels will be lower in the future compared to 2015 level. In the scenarios based on government’s plans and resident preferences segregation is higher by 15% than in the third “Global Cities” scenario. The main reason for this is that citizens prefer to settle in purely residential districts, while non-citizens prefer mixed districts and this preferred settlement trend appears to persist by 2050 in the new cities. Furthermore, the government supports this trend by offering welfare housing only in residential districts. As seen from the Global Cities scenario, which does not include any purely residential districts, but establishes mixed districts with housing, public services and business activities instead, nationality segregation may be drastically reduced. Lower segregation levels will in turn attract more investors and should contribute in transforming Kuwait into a regional trade centre according to the government’s new vision for the future.

The model results confirm that the resident responses are mostly aligned to the government’s development plans. Importantly, there are a sufficient number of people willing to migrate to the new cities. It can be concluded that establishing new cities is an effective plan as it will mitigate the traffic congestion, solve the housing shortage problems [9] and decrease the nationality segregation levels. Additionally, the new master plan will achieve lower urban density by 2050. The simulations suggest that new cities should be established in the following order: Middle region, South region, North region and finally West region. Moreover, the current urban area should continue gradual expansion by developing new mixed districts for residents who do not want to migrate to the new cities. This recommendation can be generalized and applied to other similar Gulf countries, such as Qatar and the UAE.

A major limitation of this study was that current resident preferences and responses were applied in the model as inputs for all time steps, which assumes that the responses will not change in the future. However, it was beyond the scope of research to predict and model future generation’s needs as there is a multitude of non-linear and unpredictable factors that may affect them. Future work can focus on predicting future political, social and economic conditions and integrate them in the ABM for future time steps for enhanced result reliability. In addition, further investigation about the minimum number of cities needed to reduce the negative impacts of urban growth in Kuwait can be carried out through new simulations.

## Supporting information

S1 File Survey responses and statistical tests. (DOCX)

S2 File Model description following ODD protocol. (DOCX)
