# Supplementary material for: Accounting for peoples’ preferences in establishing new cities: A spatial model of population migration in Kuwait
Source: PLoS One. 2018 Dec 13;13(12):e0209065. doi: 10.1371/journal.pone.0209065 (PMC6292647; doi:10.1371/journal.pone.0209065)
Supplement: S1 File — (DOCX) [file pone.0209065.s001.docx]

**Supplementary Material – Accounting residents’ preferences in establishing new cities: a case study of population in Kuwait**

**Nayef Alghais¹*, David Pullar¹, Elin Charles-Edwards¹**

1 School of Earth and Environmental Sciences, The University of Queensland, St Lucia, Brisbane, Queensland, Australia

***** [n.alghais@uq.edu.au](mailto:n.alghais@uq.edu.au)

# S1. Survey responses and statistical tests

The response options for Questions 1-6 were given in Likert scale, quantified with values between 0 and 5. The Likert scale responses were subject to single way ANOVA test to determine if there is a significant difference between Kuwaitis and non-Kuwaitis in their behaviours and needs. Table below shows the assigned values for each Likert scale response used in the survey analysis.

| **Response** | **Value** |
| --- | --- |
| Do not know | 0 |
| Strongly Disagree | 1 |
| Disagree | 2 |
| Neutral (Neither Agree nor Disagree ) | 3 |
| Agree | 4 |
| Strongly Agree | 5 |

For each single way ANOVA test, 0.05 was taken as the significance level value and each question was tested with a confidence level of 95%.

**Q1:** **Kuwait government is planning to develop new cities outside the existing urban area. I am considering moving to these new cities within the next 5-10 years.**

| **Answers by nationality** | | | **Response count** | **Response percent** | **Valid response percent** |
| --- | --- | --- | --- | --- | --- |
| **Kuwaitis** | Valid | 0 Do not know | 86 | 9.9 | 10.0 |
|  |  | 1 Strongly disagree | 115 | 13.2 | 13.4 |
|  |  | 2 Disagree | 124 | 14.3 | 14.4 |
|  |  | 3 Neutral | 215 | 24.8 | 25.0 |
|  |  | 4 Agree | 233 | 26.8 | 27.1 |
|  |  | 5 Strongly agree | 87 | 10.0 | 10.1 |
|  |  | Total | 859 | 98.8 | 100.0 |
|  | Missing | | 10 | 1.2 |  |
|  | **Total** | | **869** | **100.0** |  |
| **Non-Kuwaitis** | Valid | 0 Do not know | 41 | 10.8 | 11.9 |
|  |  | 1 Strongly disagree | 15 | 3.9 | 4.3 |
|  |  | 2 Disagree | 26 | 6.9 | 7.6 |
|  |  | 3 Neutral | 121 | 31.9 | 35.1 |
|  |  | 4 Agree | 98 | 25.9 | 28.6 |
|  |  | 5 Strongly agree | 43 | 11.4 | 12.5 |
|  |  | Total | 343 | 90.7 | 100.0 |
|  | Missing | | 35 | 9.3 |  |
|  | **Total** | | **378** | **100.0** |  |

| **Kuwaitis** | N | 859 |
| --- | --- | --- |
|  | Mean | 2.7617 |
|  | Std. Error of Mean | 0.05058 |
|  | Std. Deviation | 1.48226 |
|  | The 95% confidence interval of this difference | 2.6624 to 2.8609 |
| **Non-Kuwaitis** | N | 343 |
|  | Mean | 3.0185 |
|  | Std. Error of Mean | 0.07836 |
|  | Std. Deviation | 1.45207 |
|  | The 95% confidence interval of this difference | 2.8643 to 3.1726 |

*ANOVA test*

|  | **N** | **Mean** | **Std. Deviation** | **Std. Error** | **95% Confidence Interval for Mean** | |
| --- | --- | --- | --- | --- | --- | --- |
|  |  |  |  |  | **Lower Bound** | **Upper Bound** |
| **Kuwaitis** | 858 | 2.7949 | 1.43628 | .04903 | 2.6986 | 2.8911 |
| **Non-Kuwaitis** | 376 | 2.8218 | 1.54494 | .07967 | 2.6651 | 2.9785 |
| **Total** | 1234 | 2.8031 | 1.46966 | .04184 | 2.7210 | 2.8852 |

|  | **Sum of Squares** | **df** | **Mean Square** | **F** | **Sig.** |
| --- | --- | --- | --- | --- | --- |
| **Between groups** | 0.190 | 1 | 0.190 | 0.088 | 0.767 |
| **Within groups** | 2662.959 | 1232 | 2.161 |  |  |
| **Total** | 2663.148 | 1233 |  |  |  |

There were no statistically significant differences between group means (Kuwaitis and non-Kuwaitis) as determined by one-way ANOVA (*F(1,1232)*= 0.088, *p* = 0.767 > 0.05).

**Q2: I am considering moving because of financial reasons i.e. lower real estate cost or new employment opportunities. (If answer was strongly agree or agree in Question 1).**

| **Answers by nationality** | | | **Response count** | **Response percent** | **Valid response percent** |
| --- | --- | --- | --- | --- | --- |
| **Kuwaitis** | Valid | 0 Do not know | 20 | 2.3 | 6.4 |
|  |  | 1 Strongly disagree | 28 | 3.2 | 9.1 |
|  |  | 2 Disagree | 14 | 1.6 | 4.6 |
|  |  | 3 Neutral | 39 | 4.5 | 12.5 |
|  |  | 4 Agree | 142 | 16.3 | 45.6 |
|  |  | 5 Strongly agree | 68 | 7.8 | 21.8 |
|  |  | Total | 310 | 35.7 | 100.0 |
|  | Missing | | 559 | 64.3 |  |
|  | **Total** | | **869** | **100** |  |
| **Non-Kuwaitis** | Valid | 0 Do not know | 8 | 2.1 | 5.6 |
|  |  | 1 Strongly disagree | 0 | 0 | 0 |
|  |  | 2 Disagree | 3 | .7 | 1.9 |
|  |  | 3 Neutral | 17 | 4.5 | 12.1 |
|  |  | 4 Agree | 69 | 18.2 | 49.1 |
|  |  | 5 Strongly agree | 44 | 11.6 | 31.3 |
|  |  | Total | 141 | 37.1 | 100.0 |
|  | Missing | | 238 | 62.9 |  |
|  | **Total** | | **378** | **100.0** |  |

| **Kuwaitis** | N | 310 |
| --- | --- | --- |
|  | Mean | 3.4721 |
|  | Std. Error of Mean | 0.08223 |
|  | Std. Deviation | 1.44848 |
|  | The 95% confidence interval of this difference | 3.3103 to 3.6339 |
| **Non-Kuwaitis** | N | 141 |
|  | Mean | 3.9306 |
|  | Std. Error of Mean | 0.10010 |
|  | Std. Deviation | 1.18662 |
|  | The 95% confidence interval of this difference | 3.7327 to 4.1285 |

*ANOVA test*

|  | **N** | **Mean** | **Std. Deviation** | **Std. Error** | **95% Confidence Interval for Mean** | |
| --- | --- | --- | --- | --- | --- | --- |
|  |  |  |  |  | **Lower Bound** | **Upper Bound** |
| **Kuwaitis** | 316 | 3.4494 | 1.42114 | .07995 | 3.2921 | 3.6067 |
| **Non-Kuwaitis** | 142 | 3.9366 | 1.10585 | .09280 | 3.7532 | 4.1201 |
| **Total** | 458 | 3.6004 | 1.34919 | .06304 | 3.4765 | 3.7243 |

|  | **Sum of Squares** | **df** | **Mean Square** | **F** | **Sig.** |
| --- | --- | --- | --- | --- | --- |
| **Between groups** | 23.260 | 1 | 23.260 | 13.117 | 0.000 |
| **Within groups** | 808.619 | 456 | 1.773 |  |  |
| **Total** | 831.880 | 457 |  |  |  |

There was a statistically significant difference between group means (Kuwaitis and non-Kuwaitis) as determined by one-way ANOVA (*F(1,* 456*)*= 13.117, *p* = 0.000 < 0.05).

**Q3: I am considering moving because of social or other reasons i.e. to be near to relatives or friends, change of family size or to obtain free dwelling provided from the government. (If answer was strongly agree or agree in Question 1).**

| **Answers by nationality** | | | **Response count** | **Response percent** | **Valid response percent** |
| --- | --- | --- | --- | --- | --- |
| **Kuwaitis** | Valid | 0 Do not know | 7 | .8 | 2.2 |
|  |  | 1 Strongly disagree | 11 | 1.3 | 3.6 |
|  |  | 2 Disagree | 15 | 1.7 | 4.8 |
|  |  | 3 Neutral | 48 | 5.5 | 15.4 |
|  |  | 4 Agree | 176 | 20.2 | 56.7 |
|  |  | 5 Strongly agree | 54 | 6.2 | 17.3 |
|  |  | Total | 310 | 35.7 | 100.0 |
|  | Missing | | 559 | 64.3 |  |
|  | **Total** | | **869** | **100.0** |  |
| **Non-Kuwaitis** | Valid | 0 Do not know | 13 | 3.4 | 9.2 |
|  |  | 1 Strongly disagree | 3 | .9 | 2.5 |
|  |  | 2 Disagree | 12 | 3.1 | 8.4 |
|  |  | 3 Neutral | 35 | 9.3 | 25.0 |
|  |  | 4 Agree | 55 | 14.6 | 39.4 |
|  |  | 5 Strongly agree | 22 | 5.8 | 15.6 |
|  |  | Total | 141 | 37.1 | 100.0 |
|  | Missing | | 238 | 62.9 |  |
|  | **Total** | | **378** | **100.0** |  |

| **Kuwaitis** | N | 310 |
| --- | --- | --- |
|  | Mean | 3.7264 |
|  | Std. Error of Mean | 0.06032 |
|  | Std. Deviation | 1.06254 |
|  | The 95% confidence interval of this difference | 3.6077 to 3.8451 |
| **Non-Kuwaitis** | N | 141 |
|  | Mean | 3.2986 |
|  | Std. Error of Mean | 0.11784 |
|  | Std. Deviation | 1.39695 |
|  | The 95% confidence interval of this difference | 3.0656 to 3.5316 |

*ANOVA test*

|  | **N** | **Mean** | **Std. Deviation** | **Std. Error** | **95% Confidence Interval for Mean** | |
| --- | --- | --- | --- | --- | --- | --- |
|  |  |  |  |  | **Lower Bound** | **Upper Bound** |
| **Kuwaitis** | 316 | 3.7057 | 1.09780 | .06176 | 3.5842 | 3.8272 |
| **Non-Kuwaitis** | 142 | 3.3099 | 1.31647 | .11048 | 3.0915 | 3.5283 |
| **Total** | 458 | 3.5830 | 1.18280 | .05527 | 3.4744 | 3.6916 |

|  | **Sum of Squares** | **df** | **Mean Square** | **F** | **Sig.** |
| --- | --- | --- | --- | --- | --- |
| **Between groups** | 15.351 | 1 | 15.351 | 11.218 | .001 |
| **Within groups** | 623.996 | 456 | 1.368 |  |  |
| **Total** | 639.347 | 457 |  |  |  |

There was a statistically significant difference between group means (Kuwaitis and non-Kuwaitis) as determined by one-way ANOVA (*F(1,* 456*)*= 11.218, *p* = 0.001 < 0.05).

**Q4: I am not considering moving because of financial reasons i.e. living near to work or living cost is reasonable. (If answer was strongly disagree or disagree in Question 1).**

| **Answers by nationality** | | | **Response count** | **Response percent** | **Valid response percent** |
| --- | --- | --- | --- | --- | --- |
| **Kuwaitis** | Valid | 0 Do not know | 7 | .8 | 2.9 |
|  |  | 1 Strongly disagree | 13 | 1.4 | 5.5 |
|  |  | 2 Disagree | 15 | 1.7 | 6.6 |
|  |  | 3 Neutral | 26 | 3.0 | 11.4 |
|  |  | 4 Agree | 105 | 12.1 | 45.8 |
|  |  | 5 Strongly agree | 64 | 7.3 | 27.8 |
|  |  | Total | 229 | 26.3 | 100.0 |
|  | Missing | | 640 | 73.7 |  |
|  | **Total** | | **869** | **100.0** |  |
| **Non-Kuwaitis** | Valid | 0 Do not know | 0 | .1 | .8 |
|  |  | 1 Strongly disagree | 1 | .2 | 2.4 |
|  |  | 2 Disagree | 1 | .3 | 3.3 |
|  |  | 3 Neutral | 3 | .7 | 6.9 |
|  |  | 4 Agree | 18 | 4.8 | 47.6 |
|  |  | 5 Strongly agree | 15 | 3.9 | 39.0 |
|  |  | Total | 38 | 10.0 | 100.0 |
|  | Missing | | 341 | 90.0 |  |
|  | **Total** | | **378** | **100.0** |  |

| **Kuwaitis** | N | 229 |
| --- | --- | --- |
|  | Mean | 3.7521 |
|  | Std. Error of Mean | 0.08259 |
|  | Std. Deviation | 1.24865 |
|  | The 95% confidence interval of this difference | 3.5894 to 3.9149 |
| **Non-Kuwaitis** | N | 38 |
|  | Mean | 4.1504 |
|  | Std. Error of Mean | 0.15769 |
|  | Std. Deviation | 0.96958 |
|  | The 95% confidence interval of this difference | 3.8309 to 4.4700 |

*ANOVA test*

|  | **N** | **Mean** | **Std. Deviation** | **Std. Error** | **95% Confidence Interval for Mean** | |
| --- | --- | --- | --- | --- | --- | --- |
|  |  |  |  |  | **Lower Bound** | **Upper Bound** |
| **Kuwaitis** | 237 | 3.6878 | 1.29027 | .08381 | 3.5226 | 3.8529 |
| **Non-Kuwaitis** | 44 | 4.0455 | 1.05554 | .15913 | 3.7245 | 4.3664 |
| **Total** | 281 | 3.7438 | 1.26145 | .07525 | 3.5956 | 3.8919 |

|  | **Sum of Squares** | **df** | **Mean Square** | **F** | **Sig.** |
| --- | --- | --- | --- | --- | --- |
| **Between groups** | 4.748 | 1 | 4.748 | 3.005 | .084 |
| **Within groups** | 440.804 | 279 | 1.580 |  |  |
| **Total** | 445.552 | 280 |  |  |  |

There were no statistically significant differences between group means (Kuwaitis and non-Kuwaitis) as determined by one-way ANOVA (*F(1,* 279*)*= 3.005, *p* = 0.084 > 0.05).

**Q5: I am not considering moving because of social or other reasons i.e. to be near to relatives or friends or own a house/ apartment. (If answer was strongly disagree or disagree in Question 1).**

| **Answers by nationality** | | | **Response count** | **Response percent** | **Valid response percent** |
| --- | --- | --- | --- | --- | --- |
| **Kuwaitis** | Valid | 0 Do not know | 1 | .1 | .5 |
|  |  | 1 Strongly disagree | 15 | 1.8 | 6.7 |
|  |  | 2 Disagree | 9 | 1.1 | 4.0 |
|  |  | 3 Neutral | 10 | 1.1 | 4.2 |
|  |  | 4 Agree | 102 | 11.7 | 44.5 |
|  |  | 5 Strongly agree | 92 | 10.5 | 40.0 |
|  |  | Total | 229 | 26.3 | 100.0 |
|  | Missing | | 640 | 73.7 |  |
|  | **Total** | | **869** | **100.0** |  |
| **Non-Kuwaitis** | Valid | 0 Do not know | 0 | 0 | 0 |
|  |  | 1 Strongly disagree | 1 | .3 | 2.7 |
|  |  | 2 Disagree | 0 | 0 | 0 |
|  |  | 3 Neutral | 6 | 1.5 | 14.6 |
|  |  | 4 Agree | 15 | 4.0 | 39.7 |
|  |  | 5 Strongly agree | 16 | 4.3 | 43.0 |
|  |  | Total | 38 | 10.0 | 100.0 |
|  | Missing | | 341 | 90.0 |  |
|  | **Total** | | **378** | **100.0** |  |

| **Kuwaitis** | N | 229 |
| --- | --- | --- |
|  | Mean | 4.0571 |
|  | Std. Error of Mean | 0.07507 |
|  | Std. Deviation | 1.13497 |
|  | The 95% confidence interval of this difference | 3.9091 to 4.2050 |
| **Non-Kuwaitis** | N | 38 |
|  | Mean | 4.2035 |
|  | Std. Error of Mean | 0.14525 |
|  | Std. Deviation | 0.89304 |
|  | The 95% confidence interval of this difference | 3.9092 to 4.4979 |

*ANOVA test*

|  | **N** | **Mean** | **Std. Deviation** | **Std. Error** | **95% Confidence Interval for Mean** | |
| --- | --- | --- | --- | --- | --- | --- |
|  |  |  |  |  | **Lower Bound** | **Upper Bound** |
| **Kuwaitis** | 237 | 4.0127 | 1.17704 | .07646 | 3.8620 | 4.1633 |
| **Non-Kuwaitis** | 44 | 4.2045 | .87815 | .13239 | 3.9376 | 4.4715 |
| **Total** | 281 | 4.0427 | 1.13623 | .06778 | 3.9093 | 4.1761 |

|  | **Sum of Squares** | **df** | **Mean Square** | **F** | **Sig.** |
| --- | --- | --- | --- | --- | --- |
| **Between groups** | 1.366 | 1 | 1.366 | 1.059 | .304 |
| **Within groups** | 360.121 | 279 | 1.291 |  |  |
| **Total** | 361.488 | 280 |  |  |  |

There were no statistically significant differences between group means (Kuwaitis and non-Kuwaitis) as determined by one-way ANOVA (*F(1,* 279*)*= 1.059, *p* = 0.304 > 0.05).

**Q6: I prefer to reside in residential districts (Fiha, Surra or Audiliya) rather than mixed districts (Salmiya, Hawalli or Khaitan).**

| **Answers by nationality** | | | **Response count** | **Response percent** | **Valid response percent** |
| --- | --- | --- | --- | --- | --- |
| **Kuwaitis** | Valid | 0 Do not know | 5 | .6 | .7 |
|  |  | 1 Strongly disagree | 11 | 1.2 | 1.6 |
|  |  | 2 Disagree | 15 | 1.7 | 2.1 |
|  |  | 3 Neutral | 53 | 6.2 | 7.9 |
|  |  | 4 Agree | 196 | 22.6 | 28.8 |
|  |  | 5 Strongly agree | 401 | 46.1 | 58.9 |
|  |  | Total | 681 | 78.3 | 100.0 |
|  | Missing | | 188 | 21.7 |  |
|  | **Total** | | **869** | **100.0** |  |
| **Non-Kuwaitis** | Valid | 0 Do not know | 5 | 1.4 | 1.8 |
|  |  | 1 Strongly disagree | 55 | 14.5 | 18.9 |
|  |  | 2 Disagree | 110 | 29.0 | 37.9 |
|  |  | 3 Neutral | 55 | 14.6 | 19.1 |
|  |  | 4 Agree | 33 | 8.7 | 11.4 |
|  |  | 5 Strongly agree | 32 | 8.3 | 10.9 |
|  |  | Total | 289 | 76.4 | 100.0 |
|  | Missing | | 89 | 23.6 |  |
|  | **Total** | | **378** | **100.0** |  |

| **Kuwaitis** | N | 681 |
| --- | --- | --- |
|  | Mean | 4.3918 |
|  | Std. Error of Mean | 0.03548 |
|  | Std. Deviation | 0.92576 |
|  | The 95% confidence interval of this difference | 4.3222 to 4.4615 |
| **Non-Kuwaitis** | N | 289 |
|  | Mean | 2.5213 |
|  | Std. Error of Mean | 0.07490 |
|  | Std. Deviation | 1.27376 |
|  | The 95% confidence interval of this difference | 2.3738 to 2.6687 |

*ANOVA test*

|  | **N** | **Mean** | **Std. Deviation** | **Std. Error** | **95% Confidence Interval for Mean** | |
| --- | --- | --- | --- | --- | --- | --- |
|  |  |  |  |  | **Lower Bound** | **Upper Bound** |
| **Kuwaitis** | 691 | 4.4038 | .94051 | .03578 | 4.3335 | 4.4740 |
| **Non-Kuwaitis** | 319 | 2.6301 | 1.32032 | .07392 | 2.4847 | 2.7755 |
| **Total** | 1010 | 3.8436 | 1.35454 | .04262 | 3.7599 | 3.9272 |

|  | **Sum of Squares** | **df** | **Mean Square** | **F** | **Sig.** |
| --- | --- | --- | --- | --- | --- |
| **Between groups** | 686.582 | 1 | 686.582 | 594.208 | .000 |
| **Within groups** | 1164.701 | 1008 | 1.155 |  |  |
| **Total** | 1851.283 | 1009 |  |  |  |

There was a statistically significant difference between group means (Kuwaitis and non-Kuwaitis) as determined by one-way ANOVA (*F(1,* 1008*)*= 594.208, *p* = 0.000 < 0.05).

**Q7 & 8:** For these questions, a summary of the average results can be seen in below table.

| **Nationality** | **Household average size** | **Average number of servants** |
| --- | --- | --- |
| **Kuwaitis** | 7 | 2 |
| **Non-Kuwaitis** | 5 | 1 |

**Q9:** For this question, the ranking of migration criteria based on nationality can be seen in below table.

| **Criteria rank** | **Kuwaitis** | **Non- Kuwaitis** |
| --- | --- | --- |
| 1 | Lower cost of dwellings (purchase or rent). | Lower cost of dwellings (purchase or rent). |
| 2 | Closeness to the existing urban area. | Closeness to public services. |
| 3 | Closeness to public services. | Closeness to the existing urban area. |
| 4 | Closeness to government services. | Closeness to government. |
| 5 | Closeness to the sea/ beaches | Closeness to public transportation. |
| 6 | Closeness to public transportation. | Closeness to the sea/ beaches |
| 7 | Closeness to airports. | Closeness to airports. |

There is a significant difference between the two groups in terms of the criteria ranking order.

**Q 10: Please choose your first preference for settlement location based on your demands and wishes:**

| **Answers by nationality** | | | **Response count** | **Response percent** | **Valid response percent** |
| --- | --- | --- | --- | --- | --- |
| **Kuwaitis** | Valid | Stay inside the existing urban area. | 265 | 30.4 | 43.6 |
|  |  | New city in the North side of Kuwait. | 50 | 5.8 | 8.3 |
|  |  | New city in the Middle side of Kuwait. | 206 | 23.8 | 34.0 |
|  |  | New city in the West side of Kuwait. | 35 | 4.0 | 5.7 |
|  |  | New city in the South side of Kuwait. | 51 | 5.8 | 8.4 |
|  |  | Total | 607 | 69.8 | 100.0 |
|  | Missing | | 262 | 30.2 |  |
|  | **Total** | | **869** | **100.0** |  |
| **Non-Kuwaitis** | Valid | Stay inside the existing urban area. | 139 | 36.7 | 52.5 |
|  |  | New city in the North side of Kuwait. | 17 | 4.4 | 6.2 |
|  |  | New city in the Middle side of Kuwait. | 62 | 16.5 | 23.6 |
|  |  | New city in the West side of Kuwait. | 18 | 4.7 | 6.8 |
|  |  | New city in the South side of Kuwait. | 29 | 7.7 | 11.0 |
|  |  | Total | 265 | 69.9 | 100.0 |
|  | Missing | | 114 | 30.1 |  |
|  | **Total** | | **378** | **100.0** |  |

| **Kuwaitis** | N | 607 |
| --- | --- | --- |
|  | Mean | 2.2692 |
|  | Std. Error of Mean | 0.05274 |
|  | Std. Deviation | 1.29919 |
|  | The 95% confidence interval of this difference | 2.1656 to 2.3728 |
| **Non-Kuwaitis** | N | 265 |
|  | Mean | 2.1757 |
|  | Std. Error of Mean | 0.08680 |
|  | Std. Deviation | 1.41222 |
|  | The 95% confidence interval of this difference | 2.0048 to 2.3467 |

*ANOVA test*

|  | **N** | **Mean** | **Std. Deviation** | **Std. Error** | **95% Confidence Interval for Mean** | |
| --- | --- | --- | --- | --- | --- | --- |
|  |  |  |  |  | **Lower Bound** | **Upper Bound** |
| **Kuwaitis** | 625 | 2.3088 | 1.30544 | .05222 | 2.2063 | 2.4113 |
| **Non-Kuwaitis** | 293 | 2.1160 | 1.38739 | .08105 | 1.9565 | 2.2756 |
| **Total** | 918 | 2.2473 | 1.33441 | .04404 | 2.1608 | 2.3337 |

|  | **Sum of Squares** | **df** | **Mean Square** | **F** | **Sig.** |
| --- | --- | --- | --- | --- | --- |
| **Between groups** | 7.412 | 1 | 7.412 | 4.177 | .041 |
| **Within groups** | 1625.456 | 916 | 1.775 |  |  |
| **Total** | 1632.868 | 917 |  |  |  |

There was a statistically significant difference between group means (Kuwaitis and non-Kuwaitis) as determined by one-way ANOVA (*F(1,* 916*)*= 4.177, *p* = 0.041 < 0.05).

**Q11:** For this question, the most important factors chosen by responders were as below:

| **Factor type** | **Order by nationality** | |
| --- | --- | --- |
|  | **Kuwaitis** | **Non-Kuwaitis** |
| **Push factors** | High pressure on land and property values in the existing urban area. | High pressure on land and property values in the existing urban area. |
|  | Very long commuting times in the existing urban area. | Very long commuting times in the existing urban area. |
|  | Extreme housing shortages in the existing urban area. | Extreme housing shortages in the existing urban area. |
|  | High rate of accidents in the existing urban area. | High rate of accidents in the existing urban area. |
| **Pull factors** | All public services provided in the new city. | All needed public services provided in the new city. |
|  | Larger house sizes in the new city. | Ability to work in a new branch of own job in the new city. |
|  | Ability to work in a new branch of own job in the new city. | New modern train network established. |
|  | New modern train network established. | - |
