# Supplementary material for: Accounting for peoples’ preferences in establishing new cities: A spatial model of population migration in Kuwait
Source: PLoS One. 2018 Dec 13;13(12):e0209065. doi: 10.1371/journal.pone.0209065 (PMC6292647; doi:10.1371/journal.pone.0209065)
Supplement: S2 File — (DOCX) [file pone.0209065.s002.docx]

**Supplementary Material – Accounting residents’ preferences in establishing new cities: a case study of population in Kuwait**

**Nayef Alghais¹*, David Pullar¹, Elin Charles-Edwards¹**

1 School of Earth and Environmental Sciences, The University of Queensland, St Lucia, Brisbane, Queensland, Australia

***** [n.alghais@uq.edu.au](mailto:n.alghais@uq.edu.au)

# S2. Model description

The model design description presented in this section follows the ODD (Overview, Design concepts, Details) protocol for agent based models description to make the model easily as possible to be perceived by modellers [1].

## Purpose

The main objectives of the model are:

1. To simulate the residents’ migration patterns towards new cities based on their preferences and choices.
2. To investigate and predict the future impacts of urban development on nationality segregation levels via three different scenarios:
3. Government master plan scenario: Top-down development following the master plan and maintaining the existing segregation levels.
4. Residents’ preferences scenario: Bottom-up development following the resident opinions and preferences for migration, residence location and segregation, as derived from direct surveys.
5. Global cities’ scenario: Bottom-up development following the resident opinions and preferences for migration and residence location, as derived from direct surveys. However, the segregation will be simulated according to the Global Cities plan (no segregation in new cities, only mixed districts).

## Entities, State Variables and Scales

### Agents

*Decision agents (government planning authorities):* is the main decision agent responsible for establishing new cities and infrastructure.

*Resident agents*: are the agents that will be allocated to the new cities based on their preferences. They were classified into 8 different subgroups according to their age and nationality with an extra agent for the servant group (see Table 5- article).

### Environment

Districts were classified in terms of:

i) Land use in the district (residential, mixed or other use).

ii) Current population of Kuwaitis and non-Kuwaitis in each age group.

iii) Region location (existing urban area, north region, middle region, west region and south region).

### Variables

The state variable input data is spatially distributed and saved as shapefiles in vector format. The environment in this model has two levels: high level (selected scenario) and low level (the land use represented in the districts). The residents residing in a particular district (polygon), which is a part of the environment, are associated with state variables, such as nationality, age group and district settlement ID. In addition, each district has attributes with the following static state variables: ID, type (residential, mixed or other uses), maximum capacity and suitability parameters (closeness to government services, public services, sea/ beaches, the existing urban area, public transportation and airport).

The dynamic state variables are: cost of dwellings (suitability parameter), suitability weight (see Table 7- article), statutes (new or old), open date (based on the selected scenario) and population per agent group.

Data sources for the aforementioned state variables can be summarized in Table S2-1:

Table S2-1: Summary of state variables used in the model

| **Variable type** | **Content** | **Source** |
| --- | --- | --- |
| **GIS data** | Street networks, district locations and types in 2015. | Kuwait Institute for Scientific Research (KISR). |
|  | New cities data: location, capacity, districts’ types. Train network and station locations. | Kuwait municipality and interviews [2]. |
| **Demographic data** | Current population distribution on districts by nationality and age group. | Public Authority for Civil Information [3]. |
| **Population projection** | Aggregated projections from 2015-2050 by nationality and age group. | [4]. |

Other input data was obtained from the surveys, including migration ratio, preferred locations, preferred district type and household size for each agent groups (see Table 8- article).

## Initialization

Urban development of new cities in all scenarios begins in 2015. In the initialization of the model the following data will be initialized/ loaded:

1. Input demographic data: current population distribution and population projections for each agent group (nationality, age and servants).
2. Input spatial data: existing district types, street and train networks and future available cities.
3. Resident responses from the survey.

## Process Overview and Scheduling

In each 5-yearly time step after 2015, the government agent will establish new cities based on the selected scenario and then the resident agents will start migrating to the new established districts. After the end of each time step the nationality segregation level will be calculated and the output map will be updated. After reaching year 2050, the model will stop. Establishing new cities and migration modelling vary according to the selected scenario and provide different outcomes in each scenario, as seen in Table S2-2.

Table S2-2: Scenarios main differences

| **Scenario** | **Establishing a new city** | **Migration** |
| --- | --- | --- |
| **#1 Government** | Open dates, locations, number of cities and order according to the new master plan. | Based on government expectations and the suitability weights of the districts. |
| **#2 Resident** | According to master plan only for number of cities.  Predominantly based on responses obtained from the survey. | Based on the suitability weights and locations by preferred district type. |
| **#3 Global cities** |  | Based on the suitability weights and considering all new established districts are similar (mixed use). |

In the resident and global cities scenarios, opening a new city is modelled according to the state variables and after checking if there are enough residents for migrating. The model checks the responses for each resident group - young, middle and senior aged - and calculates the numbers of servants and underage residents that follow. The model establishes the new city only if there is enough interest compared to the minimum threshold, else it selects another location and repeats the process.

Before establishing a new city, infilling actions will be run for each time step in order allocate the new population in existing districts. In regards to migration to the new cities, the number of willing residents is calculated from the survey responses. The willing residents are then allocated according to their preferred district type and the suitability factors, until all districts become full or there are no more residents willing to migrate. This is done by adding new residents (points) in the new city and then deleting an equal amount of residents from the existing urban area. At the end of this cycle, the model will update the district settlement ID.

## Model flow and submodels

The model flow runs according to the following Pseudocode:

for each 5 yearly period from 2015 to 2050

infilling loop:

for all urban districts (in a random order)

if district type = residential or mixed and *Capacity* < population then

add resident agent points to district based on the average of the age distribution on the district type

Establishing new cities:

if Current_Scenario=1:

New Cities_IDs = Select Current Time Step Cities

for city in New_Cities_IDs :

result=Check_City

if result.canOpen=true:

Open_City

Else:

Writelog (“City is Faild to open this timeStep”)

elseif Current_Scenario >1:

Sort cities based on their sutabity weights

Get number should be Opened Cities= get current_Year_CitiesCount()

New Cities_IDs= Select Current remaining Cities()

for index=0 of Number should be Opened Cities :

city=New Cities_IDs_threshold[index]

for city in New_CitiesIDs_thershold:

Calculate available residents willing to migrate to the selected new city

If available residents willing to migrate to the selected new city > citiy threshold

result=Check_City

if result.canOpen=True:

Open_City

Number should be Opened Cities = Number should be Opened Cities -1

Else

Writelog (“City is Faild to open this timestep”)

Migration loop:

Select resident agent points randomly from existing urban area districts

Reallocate selected resident agent points to new city

Remove the reallocated resident agent points from existing urban area districts

Change district settlement ID for the reallocated resident agent points

Calculation of the suitability weights:

Suitability weights for cities and districts are determined by composite parameters related to proximity to government services, public services, sea/ beaches, the existing urban area, public transportation and airport and land costs. These factors are constant, except land costs that will change during the simulations, as unoccupied developable districts are converted to residential or mixed districts. The district suitability is re-computed each time step after the new land cost calculations have been finalised.

Calculation the number of residents willing to migrate to the selected new city:

This will be based on the household size for Kuwaitis and non-Kuwaitis residents by adding:

For Kuwaitis: 3 kids + 2 servants + 2 Kuwaitis

For non-Kuwaitis: 2 kids + 1 servant + 2 non-Kuwaitis.

Segregation distribution:

Distribute residents on their preferred district type. This action will be skipped in the global cities scenario; instead, in this scenario all districts will be treated as mixed and residents will be distributed based on suitability only.

Calculate the nationality segregation average:

A simple equation was used to calculate the nationality segregation at the end of each time step:

$$Nationality segregation average= \frac{Total Kuwaitis in mixed districts}{Total non-Kuwaitis in mixed district}$$

Fig S2-1 shows the model overview with all submodels.


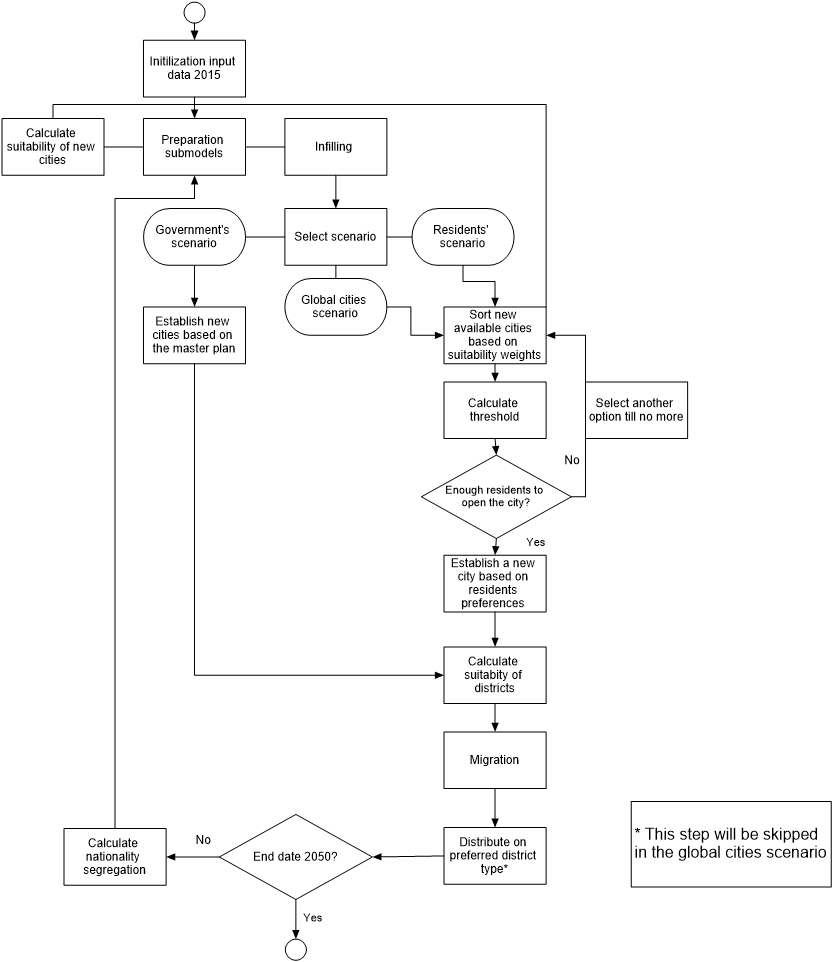


Fig S2-1: Model’s overview flowchart

## Design Concepts

- *Basic principles:* Obtaining resident opinions and applying them in simulation models will provide an opportunity to evaluate the master plan and show any differences between the government planners and residents perspectives. In addition, comparing the nationality segregation levels will provide insight about this urban issue and how it may affect transforming Kuwait into regional trade and financial centre. Finally, the resident scenario results will assess the likelihood of the occurrence of underpopulated ghost cities in Kuwait.
- *Emergence:* The key results of the model are emerging from the agent behaviours, specifically the migration patterns to the new cities, the new resident distribution and the future nationality segregation levels.
- *Adaptation:* The resident agent groups act as decision makers in the resident and global cities scenarios, in selecting the new cities to be opened according to the suitability weights and their preferred locations. This process is based on *‘if-then’* conditional decision rules. Migration to these newly opened cities is based on the suitability weights and the preferred district types (in the government and resident scenarios). In the global cities scenario, the resident agent behaviours (preferred district type) will be changed in response to environment stimuli.
- *Prediction:* Resident agent selections for their settlement district in residents and global cities scenarios are made directly from the survey input data (suitability weights and preferred locations) and after adding random sorting of the order of establishing the districts each time the model is run. The government scenario will produce only one outcome: the order of opening new cities. Additionally, the nationality segregation levels in any case will be heterogeneous.
- *Sensing:* During the migration action (allocation residents), agents based on their type are assumed to be able to sense their preferred location, district type, and suitability parameters for each district. However, they do not consider or understand the other residents’ actions nor they do determine future consequences of their selections.
- *Interactions:* Agents generally do not interact with each other. However, underage citizens and servants are linked to other agents in households for migration purposes.
- *Stochasticity:* Stochastic processes are used to simulate randomisation in the model. For example, in the resident scenario the new city selection order is randomised for migration purposes. In the global cities scenario the distribution of residents based on citizenship will be randomly determined to obtain a low nationality segregation level.
- *Collectives:* The resident agents are collectives of 100 persons of the same nationality and age group. A specific number of individuals from the collective group forms a household that is needed for migration calculations.
- *Observation:* The model code is written and recorded in Python. The variable inputs, environment and agent parameters are saved in ArcGIS database as shapefiles. In addition, the resulting maps and diagrams from the scenarios simulations at each time step are added to the same database. This provides near-perfect observation of the model in its entirety (simulations with all variables, inputs and outputs).

# References

1. Grimm, V., Berger, U., DeAngelis, D. L., Polhill, J. G., Giske, J., & Railsback, S. F. (2010). The ODD protocol: a review and first update. *Ecological modelling, 221*(23 ), 2760-2768.
2. Alghais, N., & Pullar, D. (2017). *Projection for new city futures- A case study for Kuwait*. Paper presented at the 15th International Conference on Computers in Urban Planning and Urban Management, Adelaide, Australia.

1. PACI. (2015). The total population Kuwaiti and non-Kuwaiti by age group and gender. from https://[www.paci.gov.kw/stat/SubCategory.aspx?ID=2](http://www.paci.gov.kw/stat/SubCategory.aspx?ID=2)
2. Alramadan, M., & Almusallam, M. (2013). The state of Kuwait population projection. Kuwait: Kuwait Institute for Scientific Research KISR.
